# TRIM56 acts through the IQGAP1-CDC42 signaling axis to promote glioma cell migration and invasion

**DOI:** 10.1038/s41419-023-05702-6

**Published:** 2023-03-04

**Authors:** Qing Zhang, Jianglin Zheng, Wenjie Wu, Haiyan Lian, Natasha Iranzad, Endi Wang, Lianhe Yang, Xuan Wang, Xiaobing Jiang

**Affiliations:** 1 Department of Neurosurgery, Union Hospital, Tongji Medical College, Huazhong University of Science and Technology, Wuhan, 430022 China; 2 Department of Ophthalmology, Jili Hospital of Liuyang (Liuyang Eye Hospital), Changsha, 410300 China; 3 Department of Pathology, Duke University Medical Center, Durham, NC 27710 USA; 4 Department of Pathology, The First Affiliated Hospital and College of Basic Medical Sciences, China Medical University, Shenyang, 110013 Liaoning China

**Keywords:** CNS cancer, Cell invasion

## Abstract

Diffuse invasion is an important factor leading to treatment resistance and a poor prognosis in gliomas. Herein, we found that expression of the tripartite motif containing 56 (TRIM56), a RING-finger domain containing E3 ubiquitin ligase, was markedly higher in glioma than in normal brain tissue, and was significantly correlated with malignant phenotypes and a poor prognosis. In vitro and in vivo experimental studies revealed that TRIM56 promoted the migration and invasion of glioma cells. Mechanistically, TRIM56 was transcriptionally regulated by SP1 and promoted the K48-K63-linked poly-ubiquitination transition of IQGAP1 at Lys-1230 by interacting with it, which in turn promoted CDC42 activation. This mechanism was confirmed to mediate glioma migration and invasion. In conclusion, our study provides insights into the mechanisms through which TRIM56 promotes glioma motility, i.e., by regulating IQGAP1 ubiquitination to promote CDC42 activation, which might be clinically targeted for the treatment of glioma.

## Introduction

Gliomas are the most well-known and aggressive primary intracranial tumors, characterized by an extremely disappointing prognosis [1]. Glioblastoma multiforme (GBM), which is known to be the most lethal type (WHO grade IV) of glioma in humans, accounts for ~50% of all primary central nervous system gliomas [2]. Even after optimal clinical intervention through maximal safe surgical resection followed by chemoradiotherapy, the overall survival of low-grade glioma (LGG) patients is only about 60 months, and the median survival of GBM patients is only 15 months [3]. A primary contributor to this poor prognosis is the diffuse invasive growth of glioma cells, which not only hinders complete surgical tumor resection, but also promotes chemotherapy and radiotherapy resistance [4–6]. Therefore, a comprehensive study of molecular mechanisms driving glioma migration and invasion is necessary to support the discovery of novel therapeutic targets and development of improved treatment strategies for glioma.

Malignant tumors spread through the metastasis of cancer cells to other organs via the blood and/or lymphatic vessels and through the local intra-organ invasion of cancer cells to form new lesions within the organ [5]. Although gliomas exhibit a great capacity for infiltration, they scarcely metastasize outside the brain [7]. In the brain, glioma cells migrate mainly via the perivascular space and brain parenchyma [5]. Although our knowledge of mechanisms regulating glioma cell motility has greatly improved in recent years, it remains insufficient. Therefore, further studies are required to elucidate the molecular mechanisms driving glioma migration and invasion.

Although not all tripartite motif (TRIM) family proteins containing the RING-finger domain, they are considered as E3 ubiquitin ligases and are implicated in intracellular signaling, autophagy, apoptosis, innate immunity, protein quality control, and carcinogenesis [8, 9]. A growing number of studies have confirmed that some TRIM family members are closely correlated with tumor progression. For example, TRIM24 promotes the occurrence and progression of prostate cancer by augmenting androgen receptor signaling and transcriptional activation [10]. TRIM22 inhibits osteosarcoma development by aggravating proteasomal degradation of NRF2 to motivate ROS/AMPK/mTOR/autophagy signaling, which results in autophagic cell death in osteosarcoma [11].

TRIM56, also called RNF109, harbors a RING-finger domain in the N-terminal portion, two B-box domains in the middle, and a coiled-coil domain at its C-terminus [9]. Evidence has revealed that TRIM56 plays a vital role in restricting viral activity during various infections such as those caused by influenza, yellow fever, and bovine viral diarrhea viruses, in both E3 ligase-independent as well as -dependent manner [12–14]. Additionally, TRIM56 plays distinct roles in different tumor types. In lung adenocarcinoma, hepatocellular carcinoma, and multiple myeloma, TRIM56 inhibits tumor progression by regulating the Wnt and TLR3/TRIF signaling pathways [15–17]. However, in breast cancer, TRIM56 promotes tumor progression by stabilizing ER alpha and is correlated with an unfavorable prognosis [18]. A recent study also revealed a correlation between increased TRIM56 expression and reduced tumor radio-sensitization in GBM [19]. However, the specific role of TRIM56 and the potential mechanism through which it promotes tumor progression in glioma remain incompletely understood.

Herein, we identified TRIM56 as a ubiquitin ligase that is upregulated in glioma and significantly associated with a poor prognosis. We found that TRIM56 expression was directly regulated by SP1, and TRIM56 enhanced glioma cell migration and invasion via activation of CDC42, which was mediated through the interaction of TRIM56 with IQGAP1 to increase the K48-K63-linked ubiquitination transition of IQGAP1. Therefore, targeting TRIM56 may represent a potential therapeutic approach for glioma treatment.

## Results

### TRIM56 upregulation in gliomas correlates with malignant phenotype and a poor prognosis

To determine the functions of TRIM family members in glioma, we compiled expression data of TRIM family genes and evaluated their correlation with the overall survival of glioma patients in The Cancer Genome Atlas (TCGA), CGGA_mRNA-array_301, and the Repository for Molecular Brain Neoplasia Data (REMBRANDT) datasets. Among the three datasets, high expression of TRIM5, TRIM6, TRIM21, TRIM27, TRIM28, TRIM38, TRIM45, TRIM47, and TRIM56 was significantly correlated with a poor prognosis, while the high expression of TRIM13 predicted significantly increased overall survival (Supplementary Fig. 1). In addition, we checked the TCGA database for the above-mentioned ten genes through the GEPIA portal [20] and found that only TRIM47 and TRIM56 were significantly correlated with GBM prognosis (Supplementary Fig. 2).

**Fig. 1:**
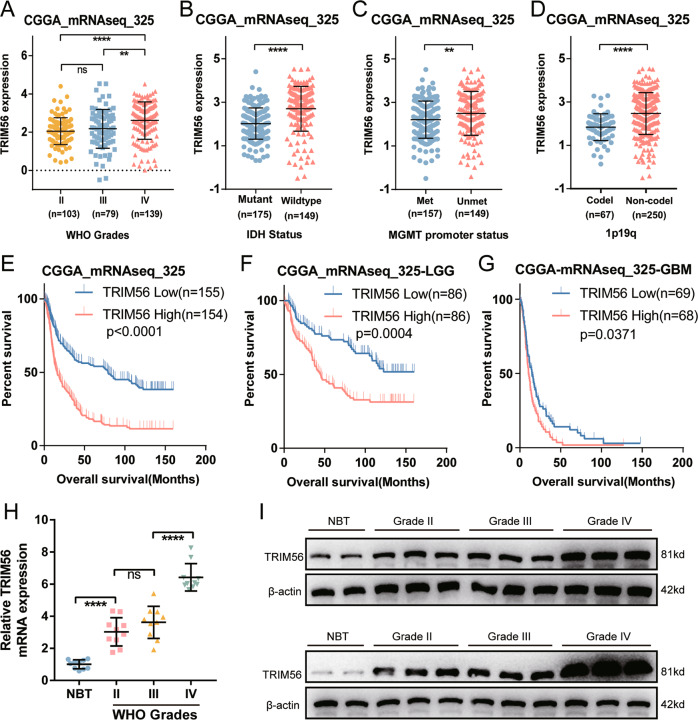
High tripartite motif containing 56 (TRIM56) expression levels correlate with malignant glioma phenotypes and a poor prognosis. **A** TRIM56 expression levels in glioma tissues from CGGA_mRNAseq_325 dataset with different WHO grades. mRNA expression levels of TRIM56 according to IDH status (**B**), MGMT promoter methylation status (**C**), and chromosome 1p19q co-deletion status (**D**) in the CGGA_mRNAseq_325 dataset (Met Methylated, Unmet Unmethylated, Codel Codeletion, Non-codel Non-codeletion). Prognostic significance of TRIM56 was identified in the CGGA_mRNAseq_325 dataset based on whole glioma (**E**), low-grade glioma (LGG) (**F**), and glioblastoma multiforme (GBM) (**G**) samples (median was the cut-off to identify high- and low-expression groups). **H** qRT-PCR analysis of TRIM56 mRNA expression in glioma specimens and normal brain tissues (NBTs) (grade II, *n* = 10; grade III, *n* = 10; grade IV, *n* = 10, NBT, *n* = 10). **I** Representative Western blots showing TRIM56 protein levels in NBTs and different grade glioma specimens.

TRIM47 promotes the proliferation, migration, and invasion of glioma cells [21–23], while the role of TRIM56 in gliomas remains unclear. Therefore, we further analyzed the expression and prognostic characteristics of TRIM56 in gliomas. Bioinformatic analyses of TRIM56 mRNA expression in the CGGA_mRNAseq_325 dataset revealed that, although the difference of TRIM56 mRNA expression was not significant between grade II and III tumors, it was remarkably higher in GBM than in LGG samples (Fig. 1A). This expression signature was also identified in the GSE16011 and REMBRANDT databases (Supplementary Fig. 3A, B). In addition, TRIM56 expression was significantly different among glioma subtypes (IDH-wild type vs. IDH-mutant, MGMT promoter methylated vs. MGMT promoter unmethylated, and Chromosome 1p19q codeletion vs. Chromosome 1p19q non-codeletion) in the CGGA_mRNAseq_325 and TCGA databases (Fig. 1B–D and Supplementary Fig. 3C–E). Furthermore, the analysis of TCGA and GTEx datasets in GEPIA revealed that TRIM56 expression was higher in both GBM and LGG samples than in normal brain tissues (NBTs) (Supplementary Fig. 3F). TRIM56 protein expression level in glioma samples have been reported to be correlated with the WHO classification of glioma grades [19]. Using a series of clinical glioma specimens, we found the same expression features of TRIM56 at both the mRNA and protein levels in glioma (Fig. 1H, I). These results confirmed that TRIM56 is expressed at significantly higher levels in glioma than in NBTs as well as in GBM than in LGG.

Considering the role of TRIM56 in glioma malignancy, its prognostic value was further assessed in both CGGA_mRNAseq_325 and TCGA cohorts. Kaplan–Meier survival analysis indicated that high TRIM56 expression was significantly associated with an unfavorable prognosis (Fig. 1E and Supplementary Fig. 3G). This prognostic feature was also noted in LGG as well as GBM samples (Fig. 1F, G and Supplementary Figs. 3H and 2J). ROC curves and area under curve (AUC) values also demonstrated the accuracy of TRIM56 in predicting the 1-, 3-, and 5-year OS of glioma, LGG, and GBM patients in CGGA_mRNAseq_325 (Supplementary Fig. 3I–K) and TCGA (Supplementary Fig. 3L–N) cohorts. Taken together, these results suggest that TRIM56 expression in gliomas is significantly increased and that this high expression significantly correlates with malignant phenotypes and a poor prognosis.

### TRIM56 is upregulated by SP1

**Fig. 2:**
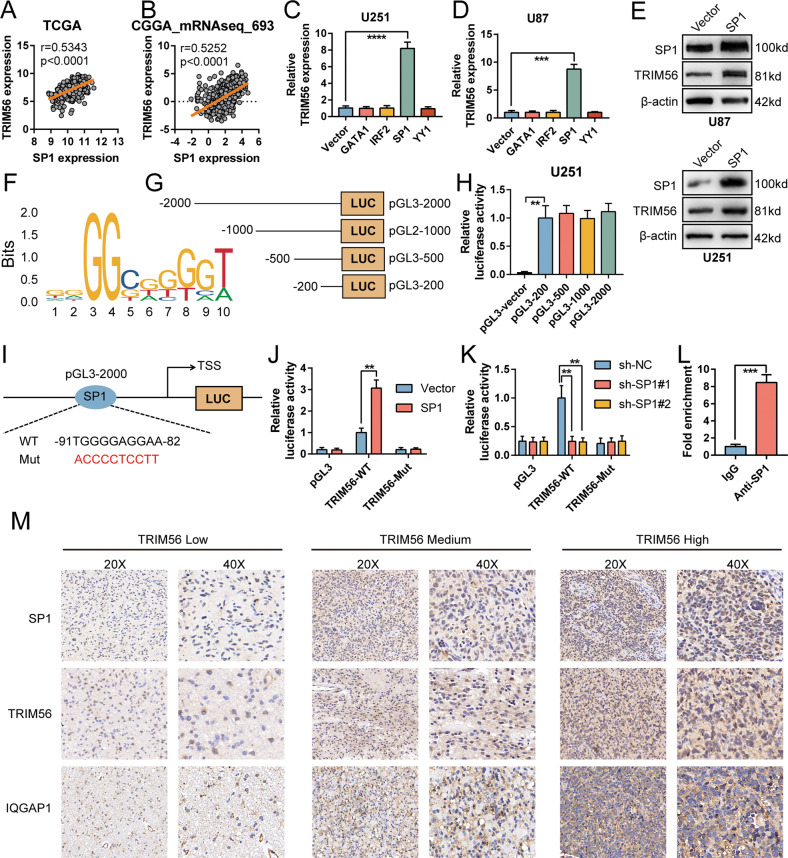
SP1 induced TRIM56 expression. Correlation between SP1 and TRIM56 expression levels in The Cancer Genome Atlas (TCGA) (**A**) and CGGA_mRNAseq_693 (**B**) cohorts. Relative TRIM56 mRNA expression levels following GATA1, IRF2, SP1, and YY1 overexpression in U251 (**C**) and U87 (**D**) cells. **E** SP1 overexpression in U87 and U251 cells upregulated TRIM56 expression. **F** The sequence motif of TRIM56 promoter for SP1 binding, analyzed via JASPAR. **G** Four truncations of the TRIM56 promoter were integrated into pGL3 plasmids. **H** Relative promoter activity of truncations in U251 cells. **I** Schematic depiction of wild type and mutant SP1 binding sites in the TRIM56 promoter. **J** Relative activity of the mutant TRIM56 promoter in SP1-overexpressing U251 cells. **K** Relative activity of the mutant TRIM56 promoter in SP1-knockdown U87 cells. **L** ChIP-qPCR analysis of SP1 bound on the promoter of TRIM56 in U251 cells. **M** Representative immunohistochemical staining displaying the relationship between TRIM56 expression and SP1, IQGAP1 expression in glioma samples, respectively.

To reveal the specific mechanism through which TRIM56 is upregulated in glioma, we examined transcription factors correlated with TRIM56 expression using the JASPAR (http://jaspar.genereg.net/), AliBaba2.1 (http://gene-regulation.com/pub/programs/alibaba2/index.html) and PROMO (http://alggen.lsi.upc.es/cgi-bin/promo_v3/promo/promoinit.cgi?dirDB=TF_8.3/) databases. GATA1, IRF2, SP1, and YY1 were screened out for further analysis. Correlation analysis in the TCGA and CGGA_mRNAseq_693 databases revealed that SP1 and IRF2 expression was notably positively correlated with that of TRIM56, while the correlation between TRIM56 and GATA1, or YY1 was not significant (Fig. 2A, B and Supplementary Fig. 4A–F). Furthermore, only SP1 overexpression led to an increase in TRIM56 expression in U87 and U251 cells (Fig. 2C, D and Supplementary Fig. 4G–Q). In contrast, SP1 knockdown inhibited TRIM56 expression in both the cell lines (Supplementary Fig. 4R, S). Western blot analysis also revealed that the overexpression and knockdown of SP1 increased and suppressed TRIM56 expression, respectively (Fig. 2E and Supplementary Fig. 4T). These results suggest that SP1 promotes TRIM56 expression at the transcriptional level. To determine the specific regulatory mechanism, we used the JASPAR database to predict the binding site of SP1 within the TRIM56 promoter region (Fig. 2F, I). We then developed a series of truncated TRIM56 promoters (Fig. 2G) and conducted luciferase reporter assays. Four truncations displayed higher promoter activities than the promoter activity of the pGL3-vector plasmid (Fig. 2H), which suggested that SP1 might have multiple binding loci within the TRIM56 promoter region or only in the −200 to 0 region. We then constructed a TRIM56 promoter mutant at −91 to −82 bp upstream of the transcription start site (TSS) (Fig. 2I). The mutant TRIM56 promoter impeded SP1 driven transcriptional activity (Fig. 2J, K), which suggested that SP1 directly bound at the -91 to -82 region of the TRIM56 promoter to activate TRIM56 transcription. ChIP-qPCR assays results also revealed that SP1 occupied the TRIM56 promoter (Fig. 2L). In addition, immunohistochemical (IHC) staining and Western blot analyses of clinical glioma tissue samples revealed a strong positive correlation between SP1 and TRIM56 expression (Fig. 2M and Supplementary Fig. 4U, W). Altogether, these results demonstrated that SP1 directly bound to the TRIM56 promoter to activate TRIM56 transcription.

### TRIM56 promotes the migration and invasion of glioma cells

**Fig. 3:**
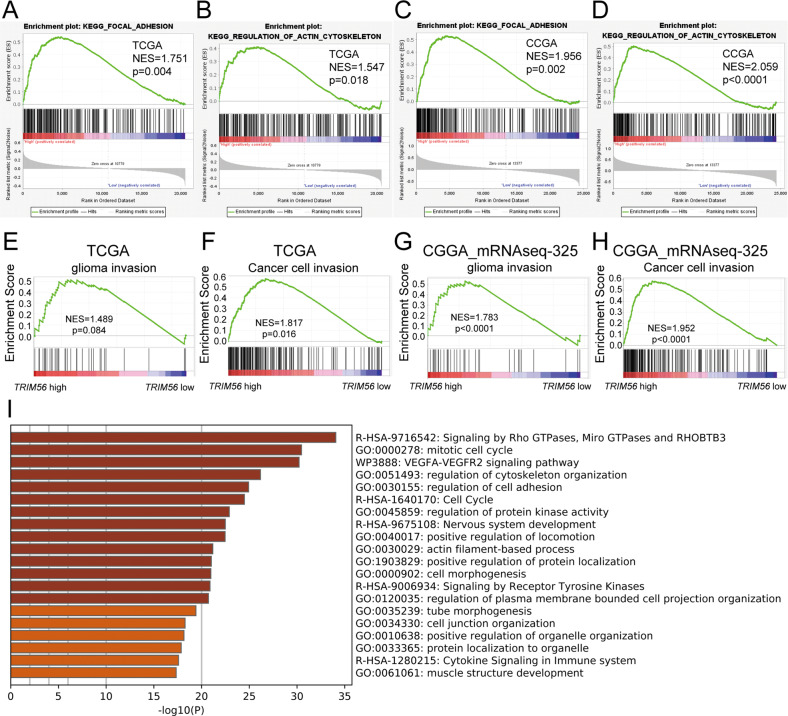
Signaling pathways significantly associated with TRIM56 in glioma. In the TCGA (**A**, **B**) and CGGA_mRNAseq_325 (**C**, **D**) cohorts, glioma samples were divided into TRIM56 high- and low-expression groups based on median TRIM56 expression. GSEA results revealed that the KEGG_FOCAL_ADHESION and KEGG_REGULATION_OF_ACTIN_CYTOSKELETON pathways were enriched in the TRIM56 high-expression group (CGGA: CGGA_mRNAseq_325 database). **E**–**H** Upon grouping the samples as described above, GSEA results revealed that glioma invasion and cancer cell invasion gene sets were enriched in the TRIM56 high-expression group. **I** Functional enrichment analysis of differentially expressed genes between TRIM56 knockdown cells and control cells in the Metascape platform.

To examine the effects of TRIM56 on glioma cell function, we first performed gene set enrichment analysis (GSEA) based on the TCGA and CGGA_mRNAseq_325 datasets. GSEA results demonstrated that high TRIM56 expression was associated with the focal adhesion and regulation of actin cytoskeleton pathways (Fig. 3A–D). Furthermore, the glioma cell invasion and cancer cell invasion gene sets were significantly enriched in the TRIM56 high-expression glioma group (Fig. 3E–H). To determine the functions of TRIM56 in gliomas with improved accuracy, we established TRIM56-knockdown and corresponding control U251 cells to explore global gene expression changes via mRNA-seq analysis. Functional enrichment analysis of significantly differentially expressed genes using Metascape [24] revealed that TRIM56 was significantly associated with signaling pathways such as signaling by Rho GTPases, Miro GTPases and RHOBTB3, regulation of cytoskeleton organization, regulation of cell adhesion, and positive regulation of locomotion (Fig. 3I). These results suggested that the signaling pathways regulated by TRIM56 were directly or indirectly related to glioma cell motility.

**Fig. 4:**
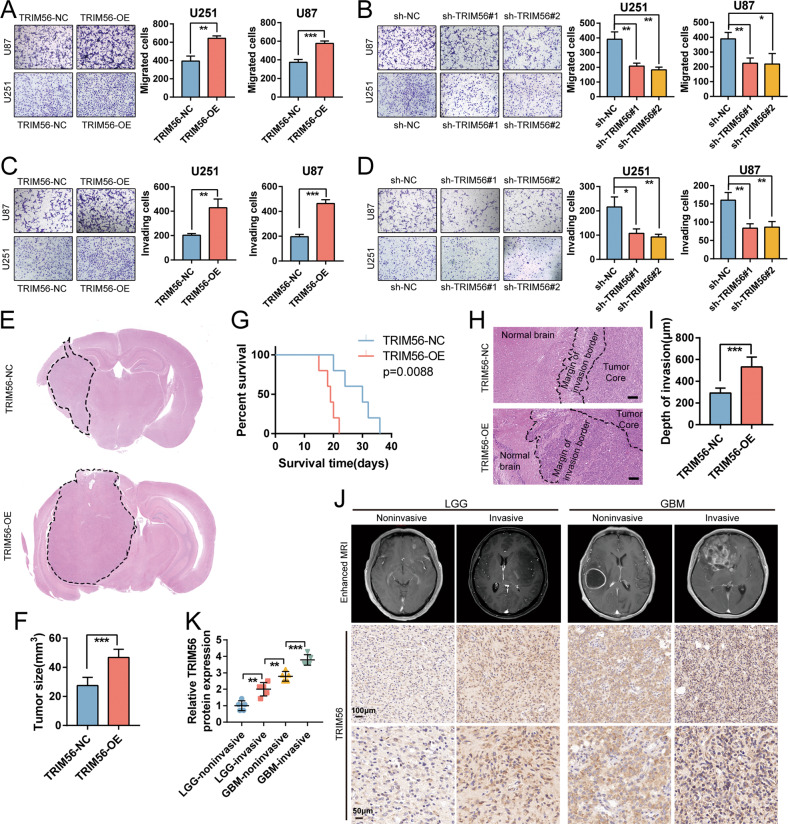
TRIM56 expression promoted glioma cell migration and invasion in vitro and in vivo. Representative images of Transwell migration assays under TRIM56 overexpression (**A**) and TRIM56 knockdown (**B**) in U87 and U251 cells and the corresponding control cells. Representative images of Transwell invasion assays under TRIM56 overexpression (**C**) and TRIM56 knockdown (**D**) in U87 and U251 cells and the corresponding control cells. **E**, **F** Representative hematoxylin and eosin (H&E) staining images showed that the sizes of xenograft tumors increased after TRIM56 overexpression. **G** Kaplan–Meier survival analysis of nude mice bearing TRIM56-NC and TRIM56-OE U87 cell-derived xenograft tumors. **H**, **I** H&E staining images showing the tumor region, normal brain region, and the transitional region. Scale bar = 100 μm. **J** Top: representative enhanced MRI images of LGG and GBM patients. Middle and bottom: low and high magnification IHC staining images of TRIM56 in glioma tissue sections. **K** Relative protein expression levels of TRIM56 detected via immunohistochemical staining in the indicated glioma samples.

Based on these results, we conducted Transwell chamber migration and invasion assays to elucidate the role of TRIM56 in these processes. As show in Fig. 4A–D, the migration and invasion abilities of U87 and U251 cells overexpressing TRIM56 were significantly enhanced (Fig. 4A, C), while TRIM56 knockdown notably impeded their migration and invasion abilities (Fig. 4B, D). The regulatory function of TRIM56 in cell motility was also observed in LN229 cells (Supplementary Fig. 5A, B). To further investigate the regulatory role of TRIM56 in glioma cell migration and invasion, we performed 3D spheroid-based invasion assays using TRIM56 knockdown and overexpressing glioma stem cells. TRIM56 overexpression promoted the invasive ability of glioma stem cells, while TRIM56 knockdown suppressed it (Supplementary Fig. 5C, D). We also developed a mouse xenograft glioma model through the intracranial transplantation of TRIM56-NC and TRIM56-OE U87 cells in nude mice. TRIM56 overexpression significantly enhanced the in vivo tumor development (Fig. 4E, F) and shortened the survival period (Fig. 4G). Interestingly, we observed a broader and more jagged tumor edge in the TRIM56-OE xenograft glioma model than in TRIM56-NC model (Fig. 4H, I), indicating that TRIM56 overexpression enhanced the invasive potential of glioma cells in vivo. Furthermore, IHC staining of glioma specimens revealed an increase in the invasive ability of glioma cells with an increase in TRIM56 expression (Fig. 4J, K). Taken together, these data indicate that TRIM56 promotes glioma cell migration and invasion.

### TRIM56 promotes glioma cell motility by promoting CDC42 activation

**Fig. 5:**
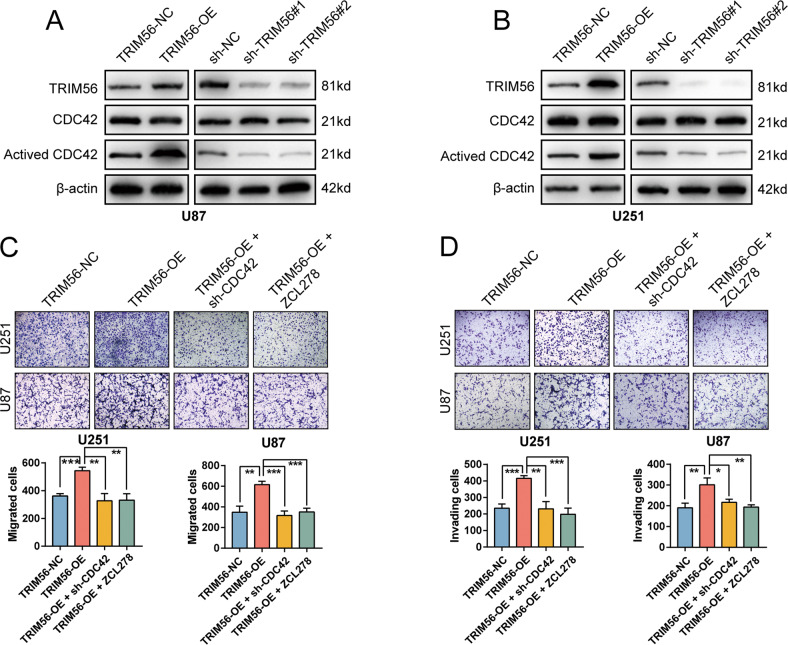
TRIM56 expression promoted glioma cell migration and invasion via CDC42 activation. In U87 (**A**) and U251 (**B**) cells, the overexpression and knockdown of TRIM56 upregulated and inhibited CDC42 activation, respectively, with neither affecting total CDC42 expression. **C** Representative images of Transwell migration assays showing that knockdown of CDC42 or treatment with the CDC42 inhibitor ZCL278 (20 μM) reversed the effect of TRIM56 overexpression on glioma cell migration. **D** Representative images of Transwell invasion assays showing that knockdown of CDC42 or treatment with the CDC42 inhibitor ZCL278 (20 μM) reversed the effect of TRIM56 overexpression on glioma cell invasion.

One of the most well-researched Rho GTPase subfamilies is CDC42 (CDC42, RHOJ, and RHOQ) [25]. Cell division cycle 42 (CDC42) plays a vital regulatory role in cellular motility and polarization, which are closely related to the cytoskeleton and cell adhesion [26–28]. Considering that the differentially expressed genes regulated by TRIM56 knockdown were highly enriched in the Rho GTPase signaling and the motility-promoting effect of TRIM56 on glioma cells, we further assessed the effect of TRIM56 on CDC42 activation. As displayed in Fig. 5A, B, TRIM56 overexpression in U87 and U251 cells resulted in an increasing in the levels of activated CDC42, whereas TRIM56 knockdown inhibited CDC42 activation. However, neither made any difference in the expression of total CDC42. To explore whether TRIM56 promoted glioma cell motility by regulating CDC42 activation, we carried out Transwell chamber migration and invasion assays on glioma cells under TRIM56 overexpression and CDC42 knockdown conditions. CDC42 knockdown or treatment with the CDC42 specific inhibitor ZCL278 [29] reversed the enhanced migration and invasion abilities of glioma cells under TRIM56 overexpression in U87, U251 and LN229 cells (Fig. 5C, D and Supplementary Fig. 6A). The 3D spheroid-based invasion assays of glioma stem cells also revealed that knockdown of CDC42 or treatment with the CDC42 specific inhibitor ZCL278 inhibited the invasion-promoting effect of TRIM56 in glioma cells (Supplementary Fig. 6B). Taken together, these results demonstrated that the positive effect of TRIM56 on glioma cell motility is mediated via CDC42 activation.

### TRIM56 protects IQGAP1 from proteasomal degradation to activate CDC42

**Fig. 6:**
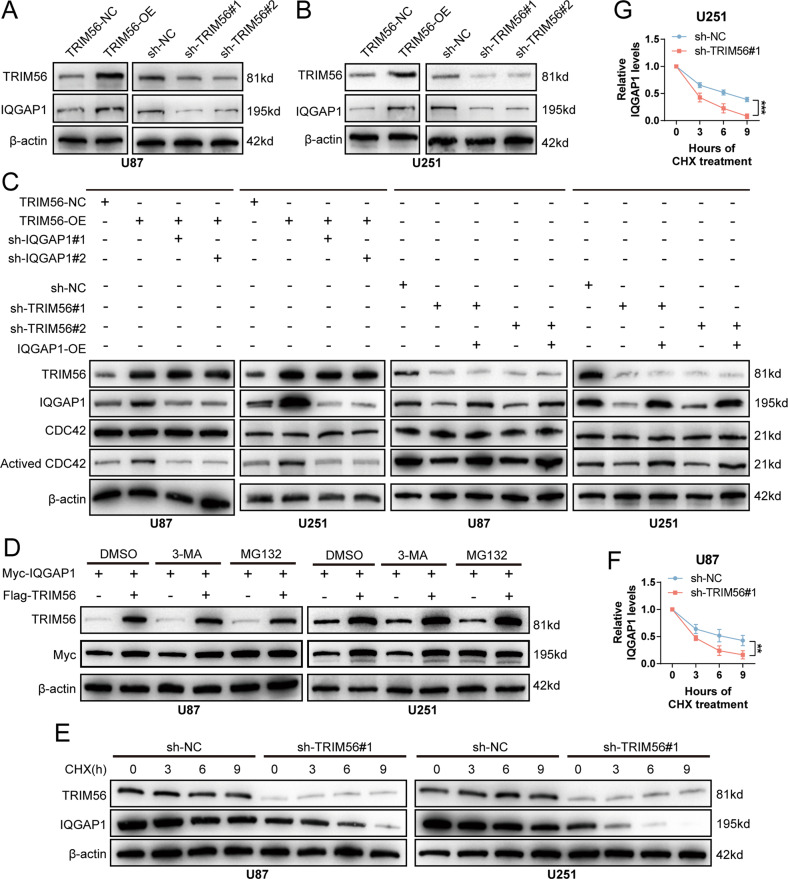
TRIM56 promoted CDC42 activation by inhibiting proteasome-mediated IQGAP1 degradation. **A**, **B** TRIM56 overexpression in glioma cells upregulated IQGAP1 expression whereas TRIM56 knockdown downregulated IQGAP1 expression. **C** In glioma cells, IQGAP1 knockdown inhibited the upregulation of CDC42 activation caused by TRIM56 overexpression, while IQGAP1 overexpression reversed the downregulation of CDC42 activation caused by TRIM56 knockdown. **D** Immunoblot assay of the lysates from U87 and U251 cells transfected with Flag-TRIM56 and Myc-IQGAP1 and then treated with DMSO, MG132 (10 μM) or 3-MA (2.5 mM). **E**–**G** In cycloheximide (CHX, 100 μg/ml)-treated cells, the degradation rate of IQGAP1 was significantly increased after TRIM56 knockdown.

CDC42 activation is directly regulated by various proteins, such as Rho guanine nucleotide exchange factor 37 (ARHGEF37) [26], IQ motif containing GTPase activating protein 1 (IQGAP1) [30–32], and N-myc downstream regulated 1 (NDRG1) [33]. In U87 and U251 cells, TRIM56 overexpression and knockdown increased and suppressed IQGAP1 expression, respectively, at protein level (Fig. 6A, B). However, neither had any effect on ARHGEF37 and NDRG1 protein levels (Supplementary Fig. 7A, B). In addition, qRT-PCR analysis demonstrated that TRIM56 knockdown had no effect on IQGAP1 mRNA expression (Supplementary Fig. 8A). IHC and Western blot analyses of clinical glioma tissue samples revealed a strong positive correlation between TRIM56 and IQGAP1 expression at the protein level (Fig. 2M and Supplementary Fig. 4V, W). In addition, IQGAP1 knockdown significantly suppressed the promoting effect of TRIM56 overexpression on CDC42 activation, while IQGAP1 overexpression reversed the inhibitory effect of TRIM56 knockdown on CDC42 activation (Fig. 6C). These results demonstrated that the regulatory effect of TRIM56 on CDC42 activation was dependent on its regulation of IQGAP1 protein expression.

TRIM56 has been shown to regulate the ubiquitination patterns of various proteins, and different ubiquitination types have different effects on protein stability [14, 34]. Considering that TRIM56 regulates IQGAP1 expression, we further assessed the effect of TRIM56 on IQGAP1 protein stability. We found that TRIM56 failed to stabilize IQGAP1 after treatment with MG132 (proteasome inhibitor), but not with 3-methyladenine (3-MA; autophagic inhibitor), which indicated that TRIM56 restrained the proteasomal degradation of IQGAP1 (Fig. 6D). In addition, following cycloheximide (CHX) treatment, TRIM56 positively modulated the turnover rate of IQGAP1 (Fig. 6E–G and Supplementary Fig. 8B, C). These results demonstrate that TRIM56 inhibits the proteasomal degradation of IQGAP1 to promote CDC42 activation.

### TRIM56 mediated the K48-K63-linked ubiquitination transition of IQGAP1 at Lys-1230

**Fig. 7:**
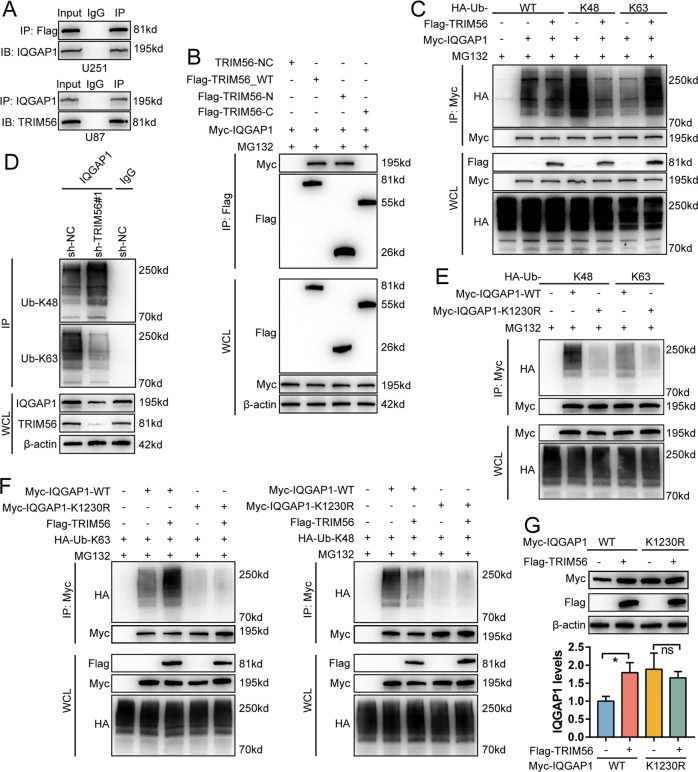
TRIM56 stabilized IQGAP1 by mediating the K48-K63 ubiquitination transition of IQGAP1. **A** Upper part, Co-IP analysis using anti-Flag antibody in U251 cells transfected with Flag-TRIM56 indicated the interaction between TRIM56 and IQGAP1; lower part: Co-IP analysis using an anti-IQGAP1 antibody revealed an interaction between IQGAP1 and TRIM56 in U87 cells. **B** Immunoprecipitation assay revealed that TRIM56_N but not TRIM56_C binding with IQGAP1 in U251 cells (MG132: 10 μM). **C** After co-transfection with the indicated plasmids in U251 cells, ubiquitinated IQGAP1 was detected via immunoprecipitation with an anti-HA antibody (MG132: 10 μM). **D** K63 and K48 ubiquitination of IQGAP1 with or without TRIM56 deficiency in U251 cells was identified via Co-IP analysis. **E** The Myc-IQGAP1-K1230R mutant abrogated the K48- and K63-linked ubiquitination of IQGAP1 (MG132: 10 μM) in U251 cells. **F** K48- and K63-linked ubiquitination of IQGAP1-K1230R was not affected by TRIM56 (MG132: 10 μM). **G** TRIM56 overexpression failed to inhibit the proteasome-mediated degradation of IQGAP1-K1230R.

Our findings indicated that TRIM56 is a RING finger domain-containing E3 ubiquitin ligase that inhibits the proteasome-mediated degradation of IQGAP1. A key process in ubiquitination mediated proteasomal degradation is the specific binding of ubiquitin ligases to their substrates. Therefore, we first examined whether there was an interaction between TRIM56 and IQGAP1. Co-IP assays indicated that TRIM56 interacted with IQGAP1 (Fig. 7A). Studies have shown that the RING-finger domain mediates the binding of TRIM56 to its substrates [35]. Thus, we established TRIM56-N (1–250 aa) (containing the RING finger domain) and TRIM56-C (251–755 aa) plasmids to determine which domain of TRIM56 is responsible for IQGAP1 binding [35]. After co-transfecting Myc-IQGAP1 with Flag-TRIM56_N, Flag-TRIM56_C, and Flag-TRIM56_WT plasmids in U251 cells, Co-IP assays revealed that TRIM56_N bound to IQGAP1 (Fig. 7B), demonstrating that the RING finger domain of TRIM56 was responsible for IQGAP1 binding.

We then assessed whether TRIM56 affects IQGAP1 ubiquitination. TRIM56 inhibited the K48-linked and promoted the K63-linked ubiquitination of IQGAP1, while having no effect on other types of ubiquitination (Fig. 7C and Supplementary Fig. 9A). Moreover, TRIM56 knockdown enhanced the K48-linked and diminished the K63-linked ubiquitination of endogenous IQGAP1, while overexpression had the opposite effect (Fig. 7D and Supplementary Fig. 9B).

Mass spectrometric analysis revealed that IQGAP1 has six ubiquitination sites (Lys-556, -1155, -1230, -1465, -1475, and -1528) [32]. Among these lysine sites, Lys-556 is situated on the coiled-coil region (CC-1K), Lys-1155 and -1230 are located in the Ras GTPase-activating protein-related domain (GRD) region of IQGAP1, while Lys-1465, -1475, and -1528 are located in the RasGAP C terminus (RGCT) region (RGCT-3K). The GRD of IQGAP1 mediates CDC42 binding [36]. It has also been shown that the ubiquitination of IQGAP1 affects its ability to regulate CDC42 activation; however, the specific mechanism through which this occurs remains unclear [32]. To identify the ubiquitination site of IQGAP1 regulated by TRIM56, we constructed IQGAP1 lysine mutants for further analysis and found that the IQGAP1 K1230R mutant simultaneously attenuated the K63- and K48-linked ubiquitination of IQGAP1 (Fig. 7E and Supplementary Fig. 9C). Furthermore, the IQGAP1 K1230R mutant abrogated the K48-K63 ubiquitination transition induced by TRIM56 (Fig. 7F). Additionally, TRIM56 failed to inhibit the proteasome-mediated degradation of IQGAP1 K1230R mutant (Fig. 7G). These results revealed that TRIM56 stabilizes IQGAP1 by inducing the K48-K63 linked ubiquitination shift of IQGAP1 at Lys-1230.

## Discussion

Emerging evidence has demonstrated that TRIM family proteins are frequently dysregulated and have versatile functions in various tumors [9, 37]. Here, we analyzed the relationship between TRIM family member expression and glioma prognosis in TCGA, CGGA_mRNA-array_301, and REMBRANDT datasets and found that TRIM56 upregulation was linked to poor prognosis in glioma patients. Previous studies have mainly focused on the functions of TRIM56 in antiviral processes [38, 39]. Although some studies have reported on TRIM56 in tumors, the specific mechanism through which it influences tumor cell function remains elusive [18]. Although previous studies have put forth that TRIM56 can suppress the radio-sensitization of GBM by regulating the FOXM1-mediated DNA repair, the function of TRIM56 in glioma has not been sufficiently studied. Our study identified TRIM56 as a glioma-favoring factor that promotes the motility of glioma cells in vitro and in vivo by potentiating CDC42 activation in an IQGAP1-dependent manner. We used mouse glioma models as well as clinical imaging data and tumor specimens to study the role of TRIM56 in glioma migration and invasion, which is highly logical and persuasive. We found that TRIM56 expression was directly transcriptionally regulated by SP1, which has not been addressed in previous studies on the role of TRIM56 in glioma [19, 40]. Mechanistically, TRIM56 was found to interact with IQGAP1 to promote its K63-linked ubiquitination and inhibit degradative K48-linked ubiquitination at Lys-1230, thus promoting CDC42 activation in glioma cells. Given that the diffuse invasiveness of glioma is closely related to chemoradiotherapy resistance [41, 42], TRIM56 may represent a promising therapeutic target in glioma.

SP1 is a well-known transcription factor that contains three highly homologous C2H2 regions [43]. SP1 is often overexpressed in several human cancers, including gastric, breast, brain, lung, pancreatic, and thyroid cancers, and promoting tumor initiation and progression [44, 45]. SP1 has been reported to promote the malignant characteristics of glioma, including invasion, cancer stem cell enrichment, and treatment-resistance [46–48]. Thus, elucidating its mechanism of action may be of great value for improving the efficiency of glioma diagnosis and treatment. As TRIM56 has been revealed to promote radiotherapy tolerance in gliomas, our finding that SP1 drives TRIM56 transcription provides evidence for the mechanism through which SP1 promotes glioma radiotherapy resistance.

As a small GTPases of the Rho family, CDC42 was first recognized for its role in establishing polarity in budding yeast [49]. CDC42 transitions between an active (GTP-bound) and an inactive (GDP-bound) state, where the former coordinates cytoskeletal processes and promotes invadopodium formation to support tumor cell invasion and dissemination, including that of glioma cells [50–52]. CDC42 activation is modulated by various factors. For example, in hepatocellular carcinoma cells, ARHGEF37 can activate CDC42 by interacting with it [26]. IQGAP1, a member of the IQGAP family, is a scaffold protein that regulates cell migration by weakening cell-cell adhesion and degrading the extracellular matrix [53–55]. Studies have confirmed that IQGAP1 phosphorylated at Ser-480 can activate CDC42 [30, 31, 56]. IQGAP1 ubiquitination also regulates its interaction with and activation of CDC42 [32]. However, the specific ubiquitination pattern of IQGAP1 and how it affects CDC42 activation remain unclear. Our study showed that TRIM56 not only interacts with IQGAP1 through the RING domain, but also inhibits its K48-linked and promotes its K63-linked poly-ubiquitination at Lys-1230, thereby inhibiting IQGAP1 degradation. This is believed to be the primary mechanism through which TRIM56 activates CDC42 to enhance glioma cell migration and invasion. Other studies on the role of TRIM56 in glioma only focused on the functional domain where TRIM56 plays a role, without clarifying the ubiquitination sites of downstream proteins regulated by TRIM56 [40].

In conclusion, we elucidated the functional role of TRIM56 in promoting the motility of glioma cells via the IQGAP1-CDC42 pathway. Considering the vital role of the IQGAP1-CDC42 pathway in cellular migration and invasion, inquiry into the TRIM56-IQGAP1 axis in glioma may open avenues for the development of novel therapeutic strategies.

## Materials and methods

### Patients and glioma tissue specimens

A total of 40 clinical glioma tissue specimens (LGG, 24 cases; GBM, 16 cases) were collected at the Neurosurgery Department of Wuhan Union Hospital from January 2020 to December 2022. Normal brain tissues (NBTs) were collected from acute brain injury patients conformed to have no prior pathologically detectable conditions. Ten LGG and ten GBM were evaluated and divided into invasive and non-invasive groups by two independent neuropathologists based on their histological and radiological characteristics (enhanced MRI images), with five cases in each group. Written informed consent was obtained from all patients associated with this study. Our study was approved by the Ethic Committee of Union Hospital of Tongji Medical College Huazhong University of Science and Technology and conducted according to the ethical principles for medical research relating to human subjects of the Declaration of Helsinki.

### Cell culture and reagents

U251 and U87 cell lines were acquired from the Cell Bank of the Chinese Academy of Sciences (Shanghai, China). LN229 cells were obtained from the American Type Culture Collection. Cells were cultured in Dulbecco’s modified Eagle’s medium (DMEM, Gibco, Grand Island, NY, USA), added with 10% fetal bovine serum (FBS) (Gibco), and incubated at 37 °C in a humidified condition supplemented with 5% CO_2_.

Patient-derived primary glioma cells were established instantly after separation of the primary patient tumor, and neurosphere cultures were conducted according to the previously published method [57, 58]. Briefly, fresh GBM tissues were disaggregated into cells using both physical and enzymatic methods and then recovered in a stem cell medium (Neurobasal-A medium with 2% B27, 20 ng/ml rh-bFGF and rh-EGF). Magnetic cell sorting was used to separate CD15^+^ GSCs from primary glioma cells. Functional analysis of self-renewal and tumor propagation were conducted to verify the cancer stem cell phenotype of extracted GSCs as described previously [59]. All cells had passed mycoplasma and the short tandem repeat (STR) DNA profiling tests.

### Immunohistochemistry (IHC) staining

After fixing with 10% neutral formalin, all specimens were chopped into serial sections at a thickness of 4 μm after being embedded in paraffin. Immunostaining was conducted via the streptavidin-peroxidase method. Briefly, following incubation with the specified primary antibody, tissue sections were processed with Elivision^TM^ Super HRP (Mouse/Rabbit) IHC Kit (Maixin, KIT-9921, Fuzhou, China) in the light of the manufacturer’s protocols. Sections were then dealt with 3,3-diaminobenzidine tetrahydrochloride (MaiXin), counterstained with hematoxylin, dehydrated in different concentrations of ethanol, and mounted with neutral gum. The protein expression level was evaluated by two independent, blinded investigators as the following intensity scores: 0 (no staining), 1 (weak staining), 2 (moderate staining), or 3 (high staining); and percentage scores: 1 (1–25%), 2 (26–50%), 3 (51–75%), and 4 (76–100%) [60]. A final score ranging from 0 to 12 was obtained by multiplying the percentage and intensity scores. Antibodies used in our study are displayed in Supplementary Table 1.

### RNA extraction and quantitative real-time reverse transcriptase PCR (qRT-PCR) analysis

TRIzol Reagent (Invitrogen, Waltham, MA, USA) was applied to isolate total RNA from tissues or cell lines. Reverse transcription was performed using Prime-Script RT Master Mix (TaKaRa, Kyoto, Japan), and qRT-PCR assays were carried out with SYBR Green Master Mix (TaKaRa) on a PCR LightCycler480 system (Roche, Basel, Switzerland). Each sample was tested in triplicate. Relative gene expression was determined based on cycle threshold (Ct) values using the 2^−ΔΔCt^ method [61]. We used β-actin as an endogenous control. Supplementary Table 2 displayed the primer sequences used in this study.

### Western blot analysis

Protein was isolated from tissues or cells and quantified using the bicinchoninic acid reagent (P0012S, Beyotime, Beijing, China). An equivalent amount of protein extracted from different samples was loaded for sodium dodecyl sulfate-polyacrylamide gel electrophoresis (SDS-PAGE) and transferred onto a PVDF membrane. After blocking with 2% bovine serum albumin, membranes were hatched with primary antibodies and thereafter with horseradish peroxidase-conjugated secondary antibodies (Proteintech, USA). Blots were detected and quantified using the Pierce™ Enhanced Chemiluminescent Substrate kit (Thermo Fisher Scientific, Waltham, MA, USA) with a ChemiDoc™ Touch detection system (Bio-Rad Laboratories, Hercules, CA, USA) and ImageJ software.

### Cell migration and invasion assays

Cell migration and invasion potential was evaluated using 24‐well Transwell chambers (8.0 µm; Corning, NY, USA), which were pre-supplemented with (Invasion assay) or without (Migration assay) 100 μl Matrigel (1:9 dilution; Coring). After the indicated treatments, cells were blended in 100 μl FBS-free DMEM and transferred to the upper chamber, while 650 μl complete DMEM supplemented with 10% FBS was added to the lower chamber. After hatching at 37 °C with 5% CO_2_ for 16–20 h, nonmigrating or non-invading cells were eliminated with a cotton tip. Paraformaldehyde (4%) was used to fix the cells moved to the lower surface of the Transwell membrane, and 0.5% crystal violet was used to stain the fixed cells. The number of stained cells was quantified under a light microscope.

### Expression plasmids and cell transfection

Flag-TRIM56 was cloned into the PGMLV-CMV-MCS-3xFlag-PGK-Puro vector. Myc-SP1 and Myc-IQGAP1 were cloned into the PLVX-MCS vector. The TRIM56 shRNA sequence was cloned into the GV112 (hU6-MCS-CMV-Puromycin) vector. The SP1, IQGAP1, and CDC42 shRNA sequences were cloned into the PLVX-shRNA-puro vector. The shRNA target sequences used for these experiments are enumerated in Supplementary Table 3. Transient transfection was performed using Lipofectamine 3000 (Invitrogen, Carlsbad, CA, USA) in the light of the manufacturer’s protocols, while stable transfection was conducted using puromycin (Sigma-Aldrich).

### RNA-sequencing (RNA-seq) analyses

Total RNA was acquired from sh-NC, sh-TRIM56#1, and sh-TRIM56#2 U251 cells. The Haplox Genomics Center (ShenZhen, China) conducted RNA‐seq library preparation and sequencing. The obtained sequence reads were aligned against the GRCh38 using HISAT2. Gene expression was analyzed using HTSeq and differential gene expression analysis was conducted using DESeq2. Differentially expressed transcripts were selected based on *p* < 0.05. The identified differentially expressed genes were subjected to functional enrichment analysis in the Metascape portal [24].

### Co‐immunoprecipitation (Co-IP) and ubiquitination assays

Immunoprecipitation assays were carried out as described previously [62]. The indicated cell lysates were hatched with specific primary antibodies and Protein A + G magnetic beads (MedChemExpress, Monmouth Junction, NJ, USA) overnight at 4 °C. Finally, after being washed with a lysis buffer, the immunocomplexes were analyzed via immunoblotting using the corresponding antibodies.

For ubiquitination analysis, cells were transfected with the specified plasmid and treated with MG132 (MedChemExpress) before treatment with lysis buffer. Immunoprecipitation was performed on cell lysates using the indicated primary antibodies. Then, the ubiquitinated proteins were hatched with protein A/G magnetic beads and detected via immunoblotting using specific primary antibodies.

### CDC42 activity assay

The CDC42 activity assay was carried out using the CDC42 Activation Assay Kit in the light the manufacturer’s protocol (Abcam, Cambridge, United Kingdom).

### Dual-luciferase assay

TRIM56 promoters were integrated into the pGL3-basic luciferase reporter plasmid. Following the pGL3 luciferase reporter plasmid, the Renilla luciferase plasmid and the other indicated plasmids were transferred into U251 cells. Dual luciferase assays were conducted in light of the manufacturer’s protocol (E1910, Promega Corporation, USA). The Renilla luciferase expression vector was used to normalize luciferase activity.

### Chromatin immunoprecipitation (ChIP) assay

ChIP assays were carried out using the ChIP Assay Kit in the light of the manufacturer’s protocol (Beyotime). In brief, after being cross-linked with formaldehyde, glioma cells were quenched with 125 mM glycine. DNA strands were sonicated into fragments of 200 to 500 bp. A SP1-specific antibody (ab231778, Abcam) and the corresponding IgG (30000-0-AP, Proteintech) were used to immuno-precipitate the cross-linked protein-DNA complexes. qRT-PCR was carried out to analyze the immunoprecipitated DNA. The primer sequences are displayed in Supplementary Table 2.

### Xenograft model

Animal experiments were performed accordance with the NIH Guidelines for the Care and Use of Laboratory Animals and were approved by the Animal Care Committee of Tongji Medical College. In brief, after being randomly divided into two groups, female BALB/c nude mice (6 weeks old, five in each group) were anesthetized, and 3 μl glioma cell (with indicated treatment) suspension (3 × 10^5^ cells) was injected into the mouse brain using a stereotaxic apparatus at 2 mm lateral and 2 mm anterior to the bregma, and at a 2 mm depth. The neurological symptoms, tumor growth, and survival time of different treated mice were carefully recorded. The tumor size was estimated using to the formula *V* = (*D* × *d*^2^)/2, where *D* stands for the longest diameter and *d* for the shortest diameter.

### Bioinformatic analysis

We assessed the expression profile of TRIM56 in The Cancer Genome Atlas (TCGA), the Repository for Molecular Brain Neoplasia Data (REMBRANDT), GSE16011, and Chinese Glioma Genome Atlas (CGGA) databases. Gene expression data and clinicopathological features of glioma samples in the TCGA database were downloaded from the official website (http://cancergenome.nih.gov/). The gene expression data and clinical information of REMBRANDT, GSE16011, CGGA_mRNAseq_325, CGGA_mRNAseq_693, and CGGA_Mrna-array_301 were acquired from the GlioVis portal (http://gliovis.bioinfo.cnio.es), Gene Expression Omnibus (GEO) database, and CGGA portal (http://www.cgga.org.cn), separately [63]. Gene set enrichment analysis (GSEA) was carried out to analyze the functional information of TRIM56 using TCGA and CGGA_mRNAseq_325 cohorts [64]. Cancer cell and glioma cell invasion related gene sets were put forward in previously published literature [65–67].

### Statistical analysis

Statistical analysis was conducted using the GraphPad Prism software (version 7.00, GraphPad; La Jolla, CA, USA) and SPSS version 25.0 (SPSS Inc., Chicago, IL, USA). An unpaired two-tailed Student’s *t* test, Wilcoxon’s test, and a one-way analysis of variance (ANOVA) were applied to compare the differences among the groups. Kaplan–Meier survival analysis was performed using log-rank tests. Pearson correlation analysis was carried out to assess gene expression correlation. All experiments were carried out at least three independent biological replicates and displayed as mean ± standard error of the mean (SEM). *p* < 0.05 was regarded as statistically significant (*p < 0.05; **p < 0.01; ***p < 0.001; ****p < 0.0001).

## Supplementary information

Supplementary Figure Legends

Supplementary Figure 1

Supplementary Figure 2

Supplementary Figure 3.

Supplementary Figure 4.

Supplementary Figure 5.

Supplementary Figure 6.

Supplementary Figure 7

Supplementary Figure 8.

Supplementary Figure 9

Supplementary Tables

reproducibility checklist

Original western blots

## Data Availability

The publicly available datasets analyzed in the current study are available in TCGA (http://can-cergenome.nih.gov/), GEO (https://www.ncbi.nlm.nih.gov/geo/), CGGA (http://www.cgga.org.cn), and GlioVis (http://gliovis.bioinfo.cnio.es/). All data generated and analyzed during this study are available upon reasonable request.
