# Supplementary material for: TRIM56 acts through the IQGAP1-CDC42 signaling axis to promote glioma cell migration and invasion
Source: Cell Death Dis. 2023 Mar 4;14(3):178. doi: 10.1038/s41419-023-05702-6 (PMC9985612; doi:10.1038/s41419-023-05702-6)
Supplement: Supplementary file 1 — Supplementary Figure Legends [file 41419_2023_5702_MOESM1_ESM.docx]

**Supplementary Figure Legends**

**Supplementary Figure 1.** **Prognostic significance of TRIM family members for glioma.**

(A-C) Survival curves showed TRIM family members (TRIM5, TRIM6, TRIM13, TRIM21, TRIM27, TRIM28, TRIM38, TRIM45, TRIM47, and TRIM56) that were significantly associated with glioma patient prognosis in both TCGA (A), CGGA_mRNA-array_301 (B) and REMBRANDT (C) databases (median was the cut-off to identify high- and low-expression groups).

**Supplementary Figure 2. Prognostic significance of TRIM family members significantly associated with glioma prognosis was analyzed in the TCGA GBM dataset.**

(A-J) Survival curves of TRIM5 (A), TRIM6 (B), TRIM13 (C), TRIM21 (D), TRIM27 (E), TRIM28 (F), TRIM38 (G), TRIM45 (H), TRIM47 (I), and TRIM56 (J) in the TCGA GBM dataset retrieved from the GEPIA portal.

**Supplementary Figure 3. Expression and prognostic characteristics of TRIM56 in glioma.**

(A, B) TRIM56 expression in different grade gliomas in the GSE16011 (A) and REMBRANDT (B) datasets. (C-E) Relationship between TRIM56 expression and IDH mutation status (C), MGMG promoter methylation status (D), and chromosome 1p19q co-deletion status (E) in the TCGA dataset. (F) TRIM56 expression in GBM, LGG, and NBTs were analyzed by integrating TCGA and GTEx databases using GEPIA. (G-H) Survival curves of TRIM56 in TCGA glioma (G) and LGG (H) datasets retrieved from the GEPIA portal. (I-N) Receiver operating characteristic (ROC) curve analysis evaluating the performance of TRIM56 in predicting the 1-, 3- and 5-year OS of whole glioma, LGG and GBM samples from CGGA_mRNAseq_325 (I-K) and TCGA (L-N) cohorts.

**Supplementary Figure 4. SP1 regulated TRIM56 transcription.**

(A, B) Correlation analysis between GATA1 and TRIM56 expression levels in TCGA (A) and CGGA_mRNAseq_693 (B) datasets. (C, D) Correlation analysis between IRF2 and TRIM56 expression levels in TCGA (C) and CGGA_mRNAseq_693 (D) datasets. (E, F) Correlation analysis between YY1 and TRIM56 expression levels in TCGA (E) and CGGA_mRNAseq_693 (F) datasets. (G-J) Detection of GATA1 (G), IRF2 (H), SP1 (I), and YY1 (J) overexpression efficiency in U251 cells via qRT-PCR. (K-N) Detection of GATA1 (K), IRF2 (L), SP1 (M), and YY1 (N) overexpression efficiency in U87 cells via qRT-PCR. (O-Q) The overexpression efficiencies of GATA1 (O), IRF2 (P), and YY1 (Q) in U251 and U87 cells were detected via Western blot. (R) Detection of SP1 knockdown efficiency in U251 and U87 cells via qRT-PCR. (S) The effect of SP1 knockdown in U251 and U87 on TRIM56 expression was assessed via qRT-PCR. (T) SP1 knockdown in U87 and U251 cells reduced the protein expression of TRIM56. (U-V) Immunohistochemical staining analysis of glioma samples found that the expression levels of TRIM56 were significantly positively correlated with those of SP1 (U) and IQGAP1 (V). (W) Western blot analysis revealed the correlation of TRIM56 and SP1 as well as TRIM56 and IQGAP1 expression levels in glioma specimens.

**Supplementary Figure 5. TRIM56 promoted glioma cell motility.**

(A-B) Transwell migration (A) and invasion (B) assays of LN229 cells after TRIM56 overexpression or knockdown. (C-D) Representative images of the 3D spheroid invasion assay of TRIM56 overexpression (C) and knockdown (D) in GBM01 cell and the corresponding control cells.

**Supplementary Figure 6. TRIM56 promoted glioma cell motility by activating CDC42.**

(A) Knockdown of CDC42 or treatment with the CDC42-specific inhibitor ZCL278 (20 μM) blocked the promotion of LN229 cell migration and invasion by TRIM56 overexpression. (B) Knockdown of CDC42 or treatment with the CDC42 inhibitor ZCL278 (20 μM) inhibited the effect of TRIM56 overexpression on GBM01 invasion.

**Supplementary Figure 7. TRIM56 did not affect the expression of ARHGEF37 and NDRG1**.

(A) Overexpression and knockdown of TRIM56 in U251 cells did not affect the expression of ARHGEF37 and NDRG1. (B) Overexpression and knockdown of TRIM56 in U87 cells did not affect the expression of ARHGEF37 and NDRG1.

**Supplementary Figure 8. TRIM56 inhibited the proteasome-mediated degradation of IQGAP1.**

(A) TRIM56 knockdown did not affect the mRNA expression of IQGAP1. (B, C) The degradation rate of IQGAP1 was significantly reduced after TRIM56 overexpression in CHX (100 μg/ml) treated U87 and U251 cells.

**Supplementary Figure 9. TRIM56 regulated the ubiquitination patterns of IQGAP1.**

(A) Ubiquitination analysis performed using extracts of U251 cells transfected with indicated plasmid. (B) TRIM56 overexpression in U251 cells increased the K48-K63-linked ubiquitination transition of endogenous IQGAP1. (C) Co-IP and immunoblot analyses of MG132 (10 μM) treated U251 cell extracts following transfection with Myc-IQGAP1 wild-type (WT) or indicated mutants (CC-1K: K556R; K1155: K1155R; K1230: K1230R; RGCT-3K: K1465R, K1475R, and K1528R) using the corresponding antibody.
