# Supplementary figures and images for: TRIM56 acts through the IQGAP1-CDC42 signaling axis to promote glioma cell migration and invasion
Source: Cell Death Dis. 2023 Mar 4;14(3):178. doi: 10.1038/s41419-023-05702-6 (PMC9985612; doi:10.1038/s41419-023-05702-6)

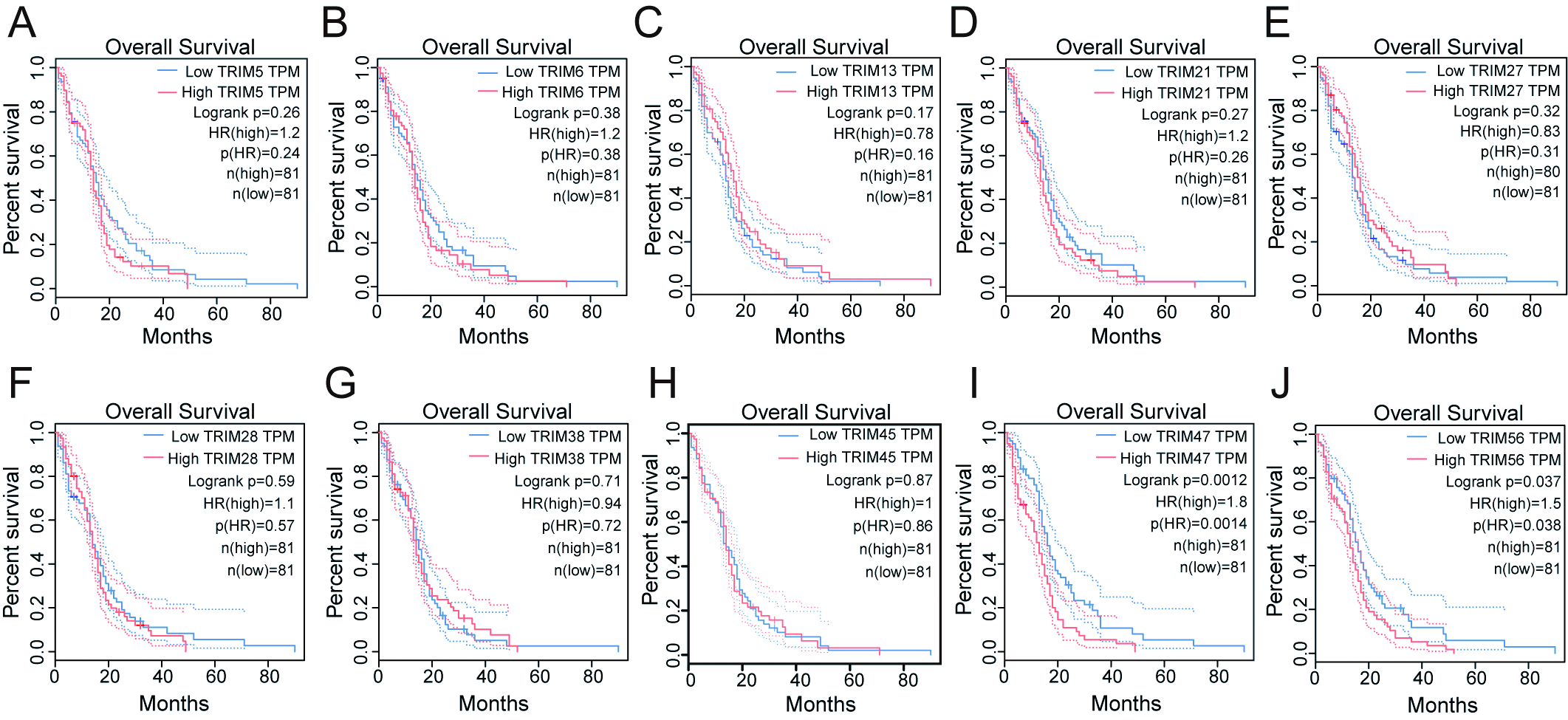

Supplement: Supplementary file 3 — Supplementary Figure 2 [file 41419_2023_5702_MOESM3_ESM.tif]

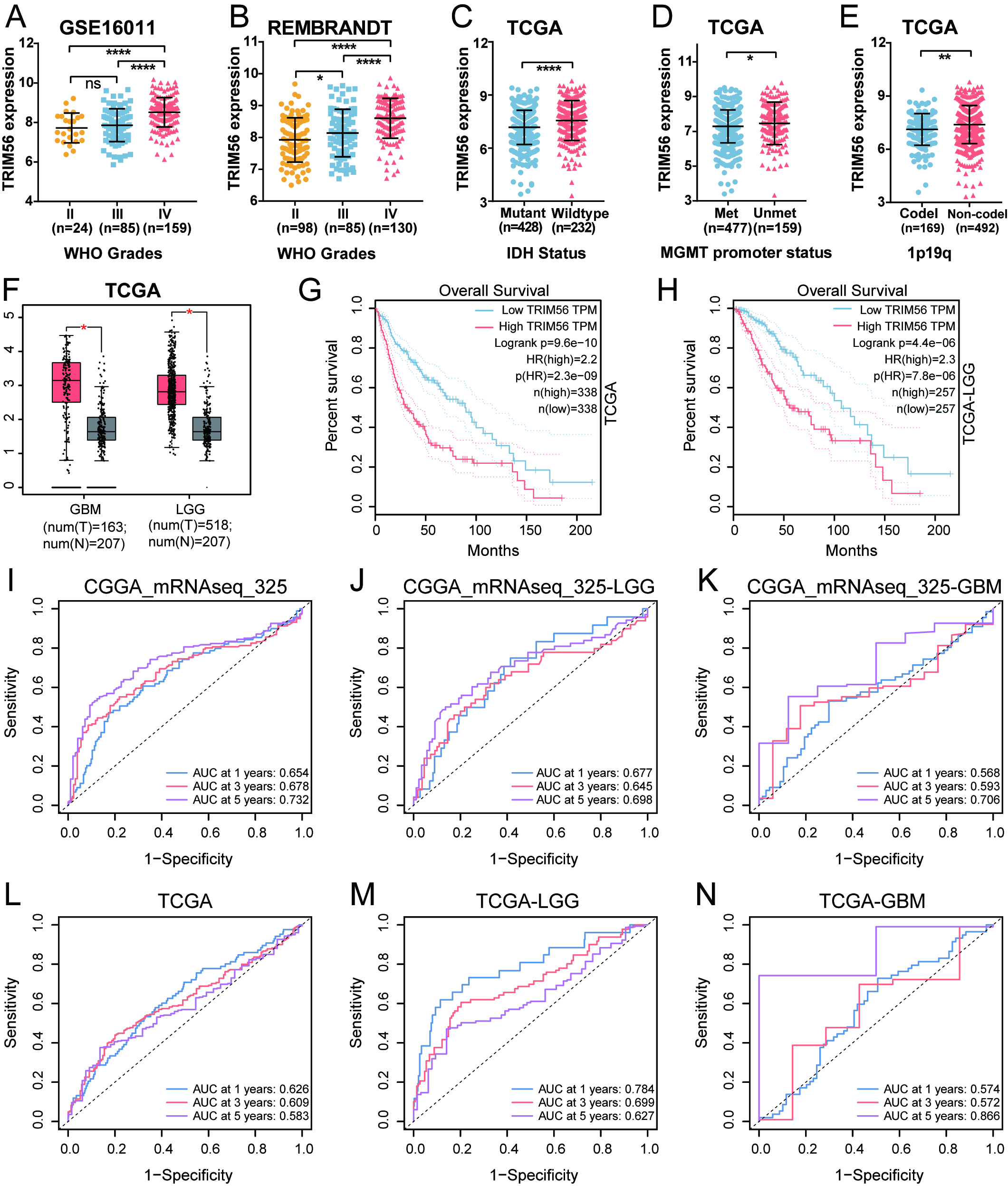

Supplement: Supplementary file 4 — Supplementary Figure 3. [file 41419_2023_5702_MOESM4_ESM.tif]

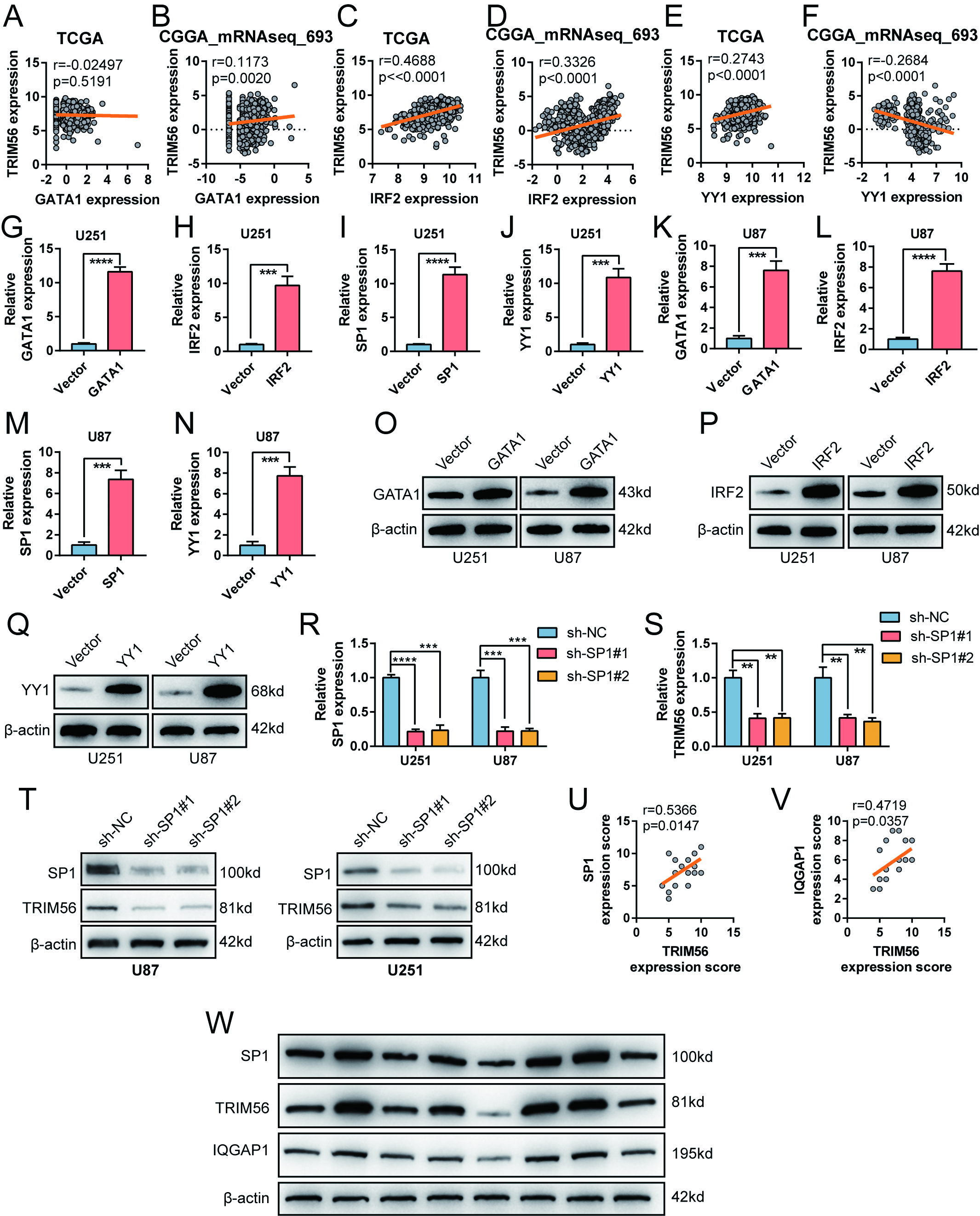

Supplement: Supplementary file 5 — Supplementary Figure 4. [file 41419_2023_5702_MOESM5_ESM.tif]

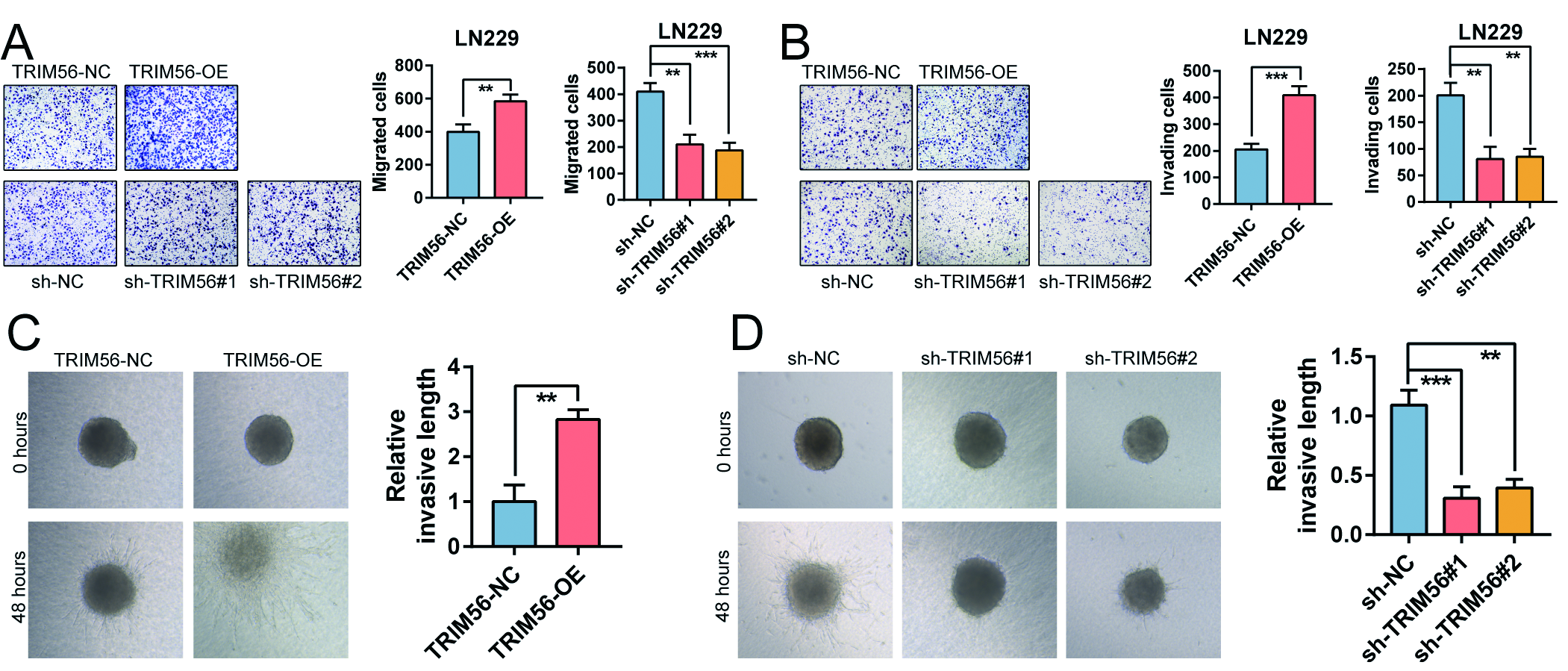

Supplement: Supplementary file 6 — Supplementary Figure 5. [file 41419_2023_5702_MOESM6_ESM.tif]

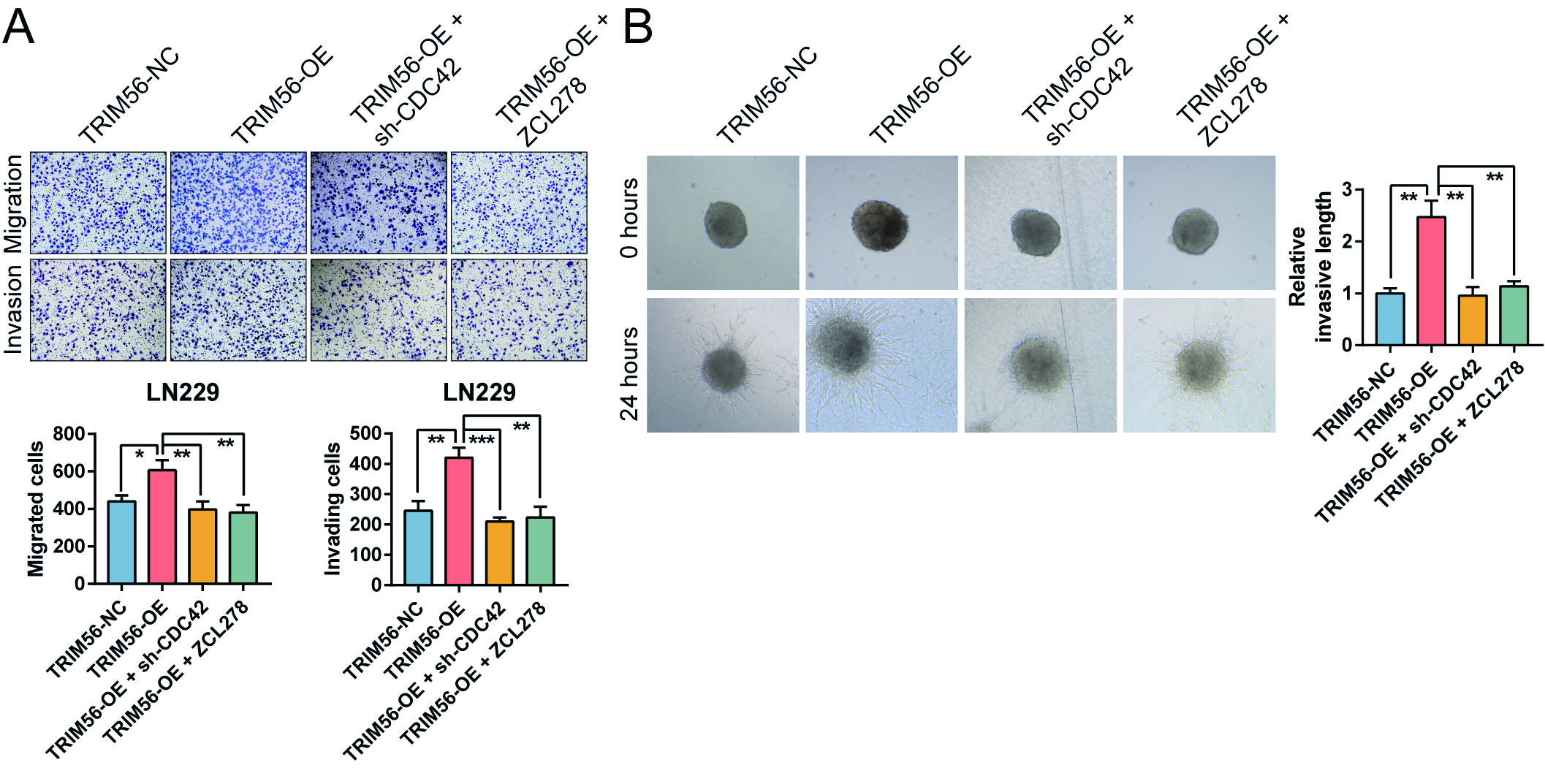

Supplement: Supplementary file 7 — Supplementary Figure 6. [file 41419_2023_5702_MOESM7_ESM.tif]

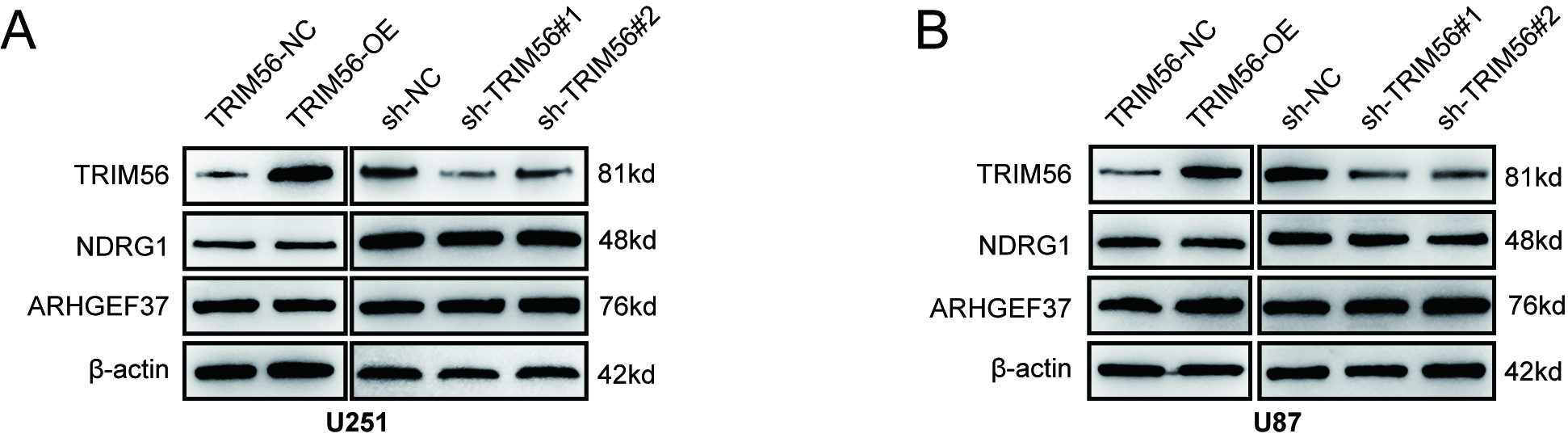

Supplement: Supplementary file 8 — Supplementary Figure 7 [file 41419_2023_5702_MOESM8_ESM.tif]

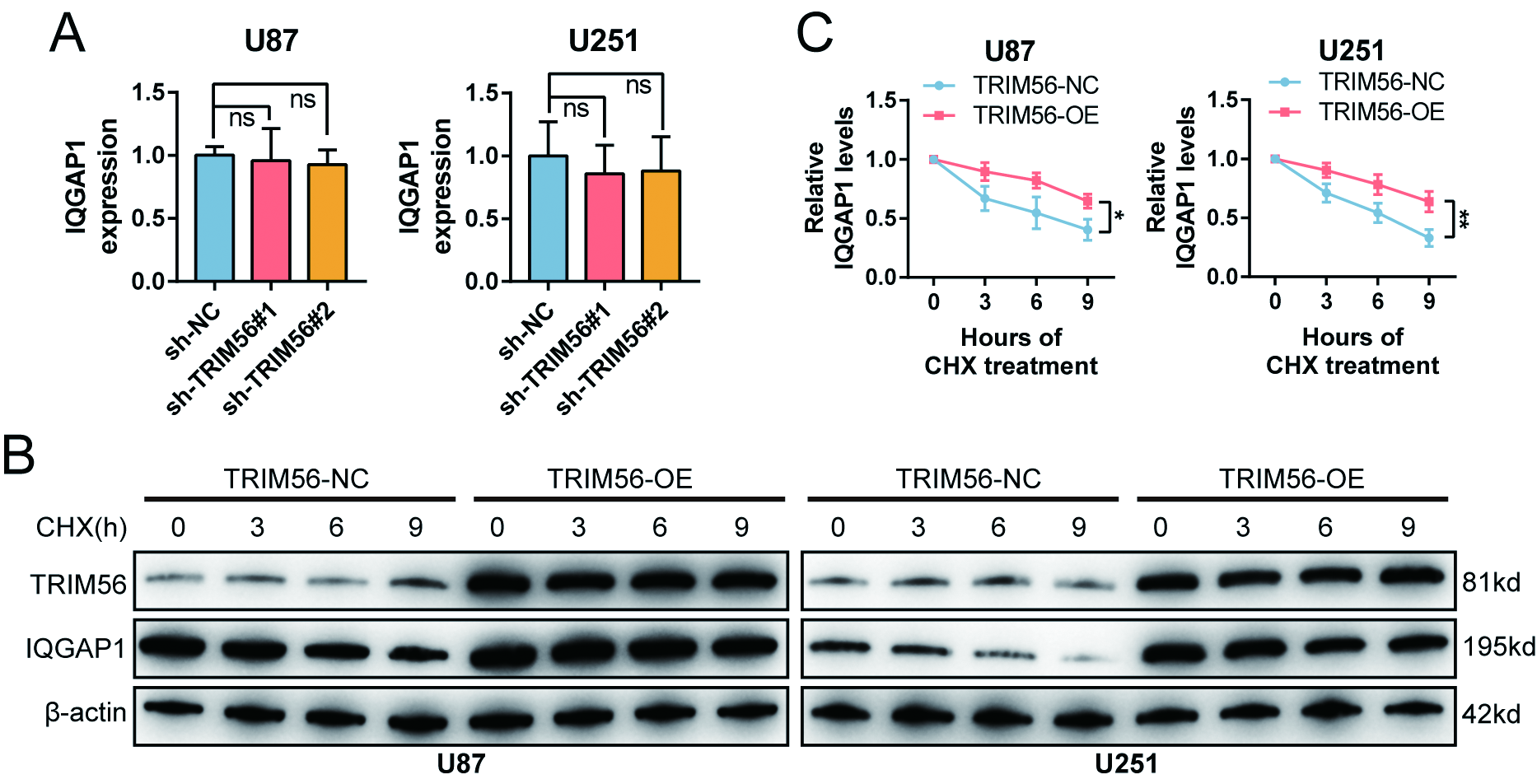

Supplement: Supplementary file 9 — Supplementary Figure 8. [file 41419_2023_5702_MOESM9_ESM.tif]

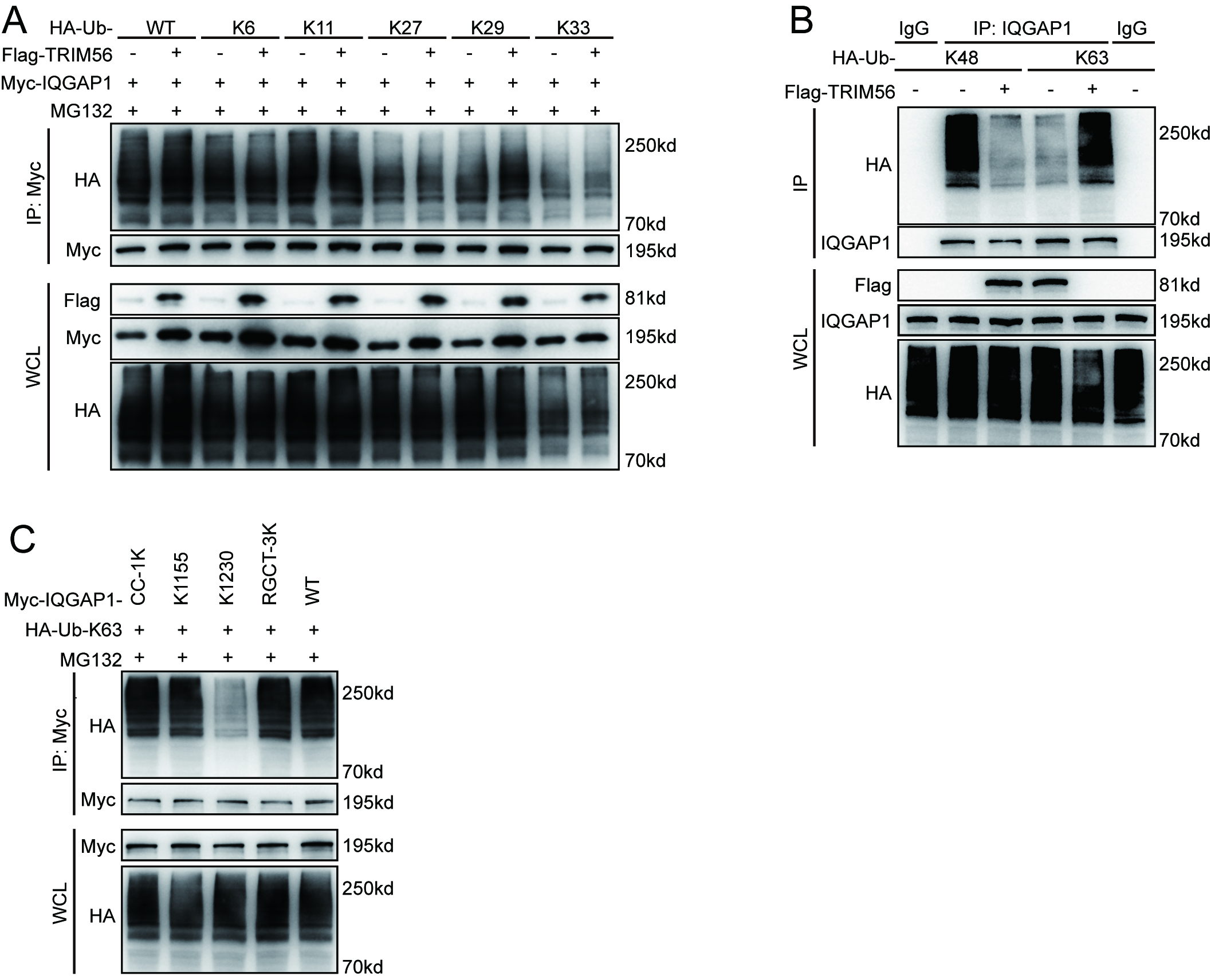

Supplement: Supplementary file 10 — Supplementary Figure 9 [file 41419_2023_5702_MOESM10_ESM.tif]
