# Supplementary material for: TRIM56 acts through the IQGAP1-CDC42 signaling axis to promote glioma cell migration and invasion
Source: Cell Death Dis. 2023 Mar 4;14(3):178. doi: 10.1038/s41419-023-05702-6 (PMC9985612; doi:10.1038/s41419-023-05702-6)
Supplement: Supplementary file 11 — Supplementary Tables [file 41419_2023_5702_MOESM11_ESM.pdf]

Supplementary Table 1：Lists of antibodies used in this study.

Western Blot

Antibody	Company name	Dilution	Catalogue number
Anti-TRIM56 antibody	Abcam	1:10000	ab154862
Beta Actin Monoclonal antibody	Proteintech	1:20000	66009-1-Ig
Anti-SP1 antibody	Abcam	1:1000	ab231778
IQGAP1 Polyclonal antibody	Proteintech	1:2000	22167-1-AP
CDC42 Rabbit pAb	Abclone	1:1000	A1188
MYC tag Monoclonal antibody	Proteintech	1:2000	60003-2-Ig
DYKDDDDK tag Monoclonal antibody (Binds to FLAG® tag epitope)	Proteintech	1:5000	66008-4-Ig
GATA1 Rabbit pAb	Abclone	1:1000	A0475
IRF2 Rabbit pAb	Abclone	1:1000	A2558
YY1 Rabbit pAb	Abclone	1:1000	A12928

Immunohistochemistry

Antibody	Company name	Dilution	Catalogue number
Anti-TRIM56 antibody	Abcam	1/1000	ab154862
Anti-SP1 antibody	Abcam	1:500	ab231778
IQGAP1 Polyclonal antibody	Proteintech	1:100	22167-1-AP

Supplementary Table 2: PCR Primers sequences.

qRT-PCR Primers

Gene name	Forward (5′-3′)	Rerverse (5′-3′)
TRIM56	TCTTCGGCTGTCCTTTGAGGAG	CTGTCTCTCAGTCTTCGGCTCA
β-actin	TGACGTGGACATCCGCAAAG	CTGGAAGGTGGACAGCGAGG
GATA1	GCTTCCTGGAGACTTTGA	TACCTGCCCGTTTACTGA
IRF2	AACTGGGCAATCCATACA	TGAAGTCAGGACCGCATA
SP1	CAGCCCAGATGCCCAACC	GCCCCTTCCTTCACTGTCTTTA
YY1	AAGTGGGAGCAGAAGCAG	CCAGTTGGTGTCGTTTTAG
IQGAP1	TTATCACCCTCATTCGTT	GACCTCCCTTCTGTTTATT

ChIP qRT-PCR Primers

TRIM56	CCGTGTCTGTGGATAAGA	GGTAGGCTGTCAGGAAGT

Supplementary Table 3: Lists of target sequences of shRNA used in this study.

shRNA

shRNA	Vector	Target sequences
sh-TRIM56#1	hU6-MCS-CMV-Puro	gcAGCAGAATAGTGTGGTAAT
sh-TRIM56#2	hU6-MCS-CMV-Puro	cgCACGGCTCTATCTCATCAA
sh-SP1#1	PLVX-shRNA-puro	GCGTTTCTGCAGCTACCTTGA
sh-SP1#2	PLVX-shRNA-puro	GCAGACCTTTACAACTCAAGC
sh-IQGAP1#1	PLVX-shRNA-puro	GCATCCACTTACCAGGATATA
sh-IQGAP1#2	PLVX-shRNA-puro	GCAGAAGAAGGCATATCAAGA
sh-CDC42#1	PLVX-shRNA-puro	GCTTGTTGGGACTCAAATTGA
