# Supplementary material for: TRIM56 acts through the IQGAP1-CDC42 signaling axis to promote glioma cell migration and invasion
Source: Cell Death Dis. 2023 Mar 4;14(3):178. doi: 10.1038/s41419-023-05702-6 (PMC9985612; doi:10.1038/s41419-023-05702-6)

Fig 1I

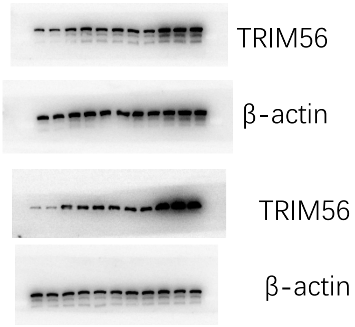

Fig 2

Fig 2E

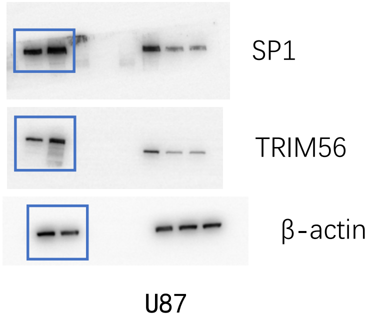

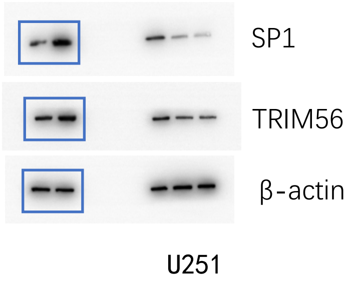

Fig 5

Fig5 A

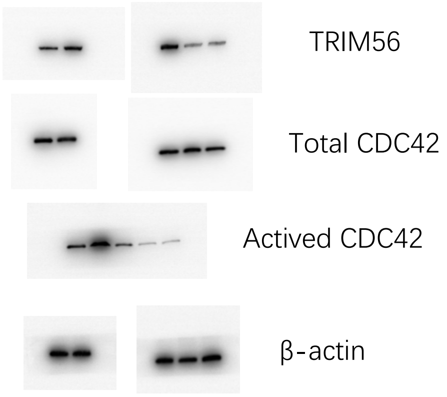

Fig 5B

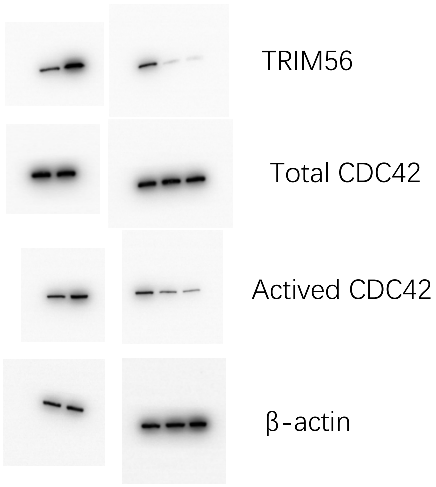

Fig 6

Fig 6A

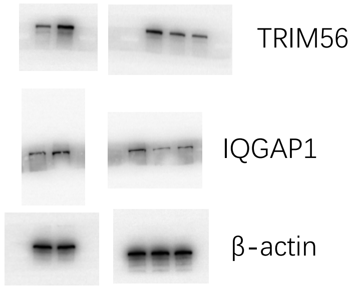

Fig 6B

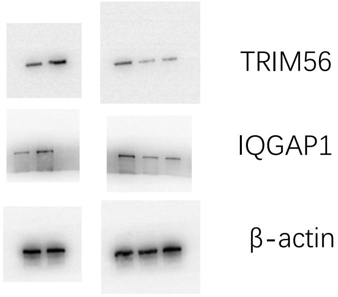

Fig 6C

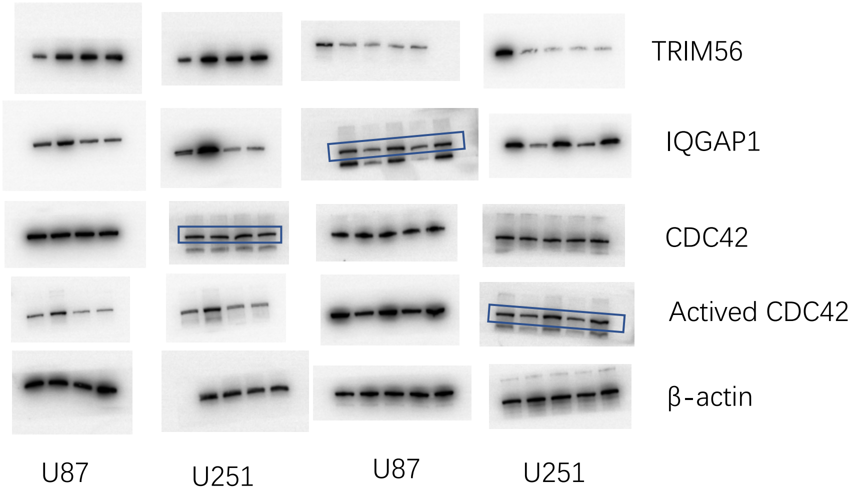

Fig 6D

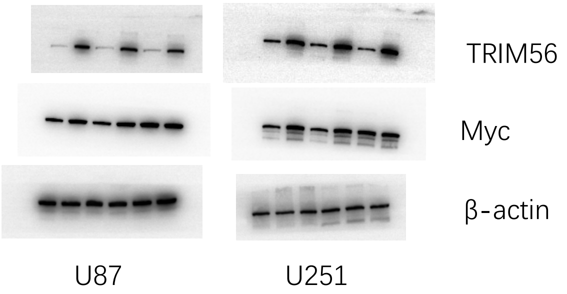

Fig 6E

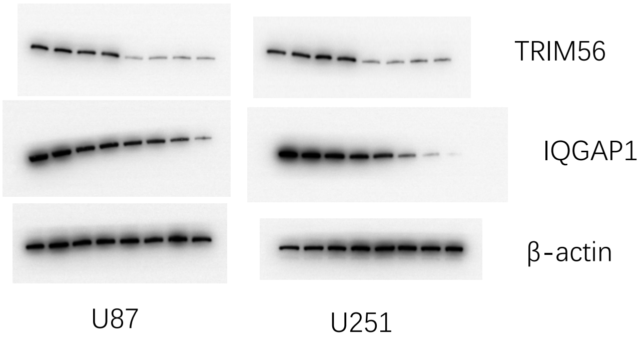

Fig 7

Fig 7A

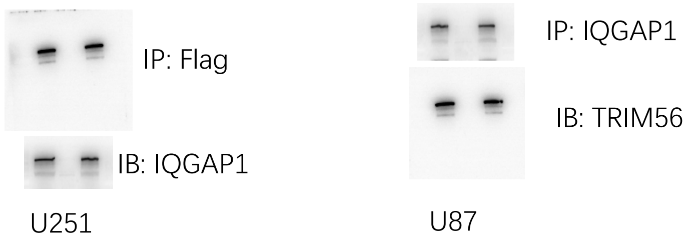

Fig 7B

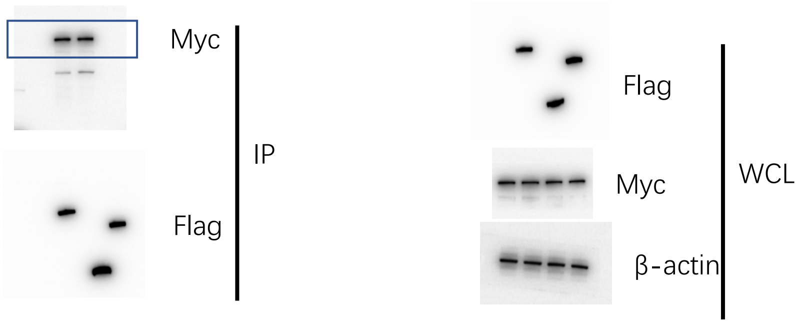

Fig 7C

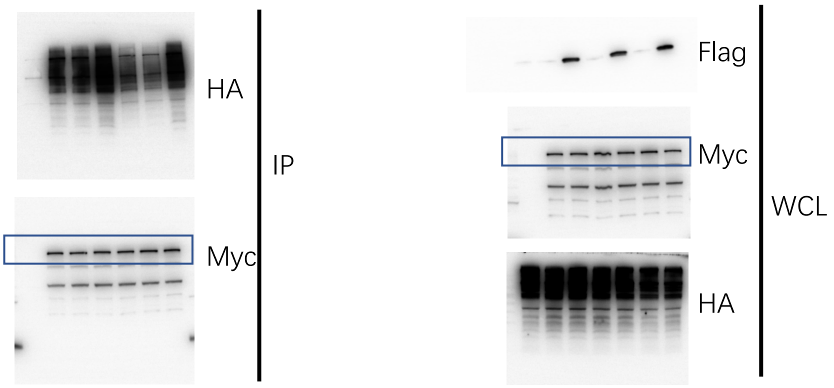

Fig 7D

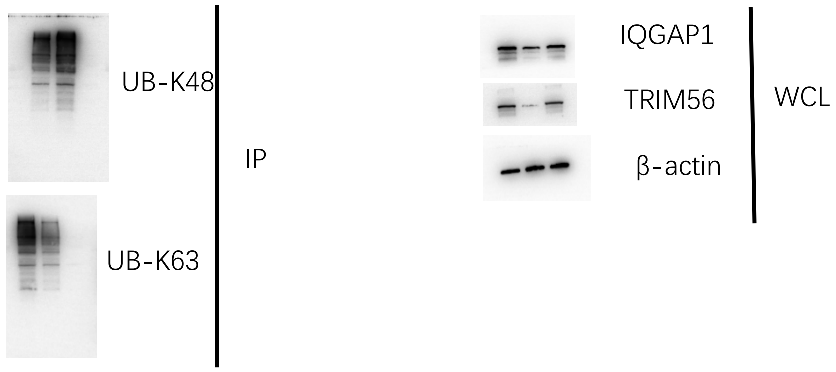

Fig 7E

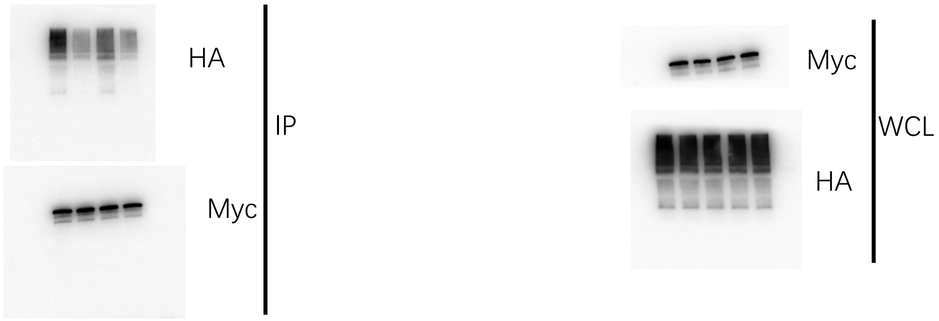

Fig 7F

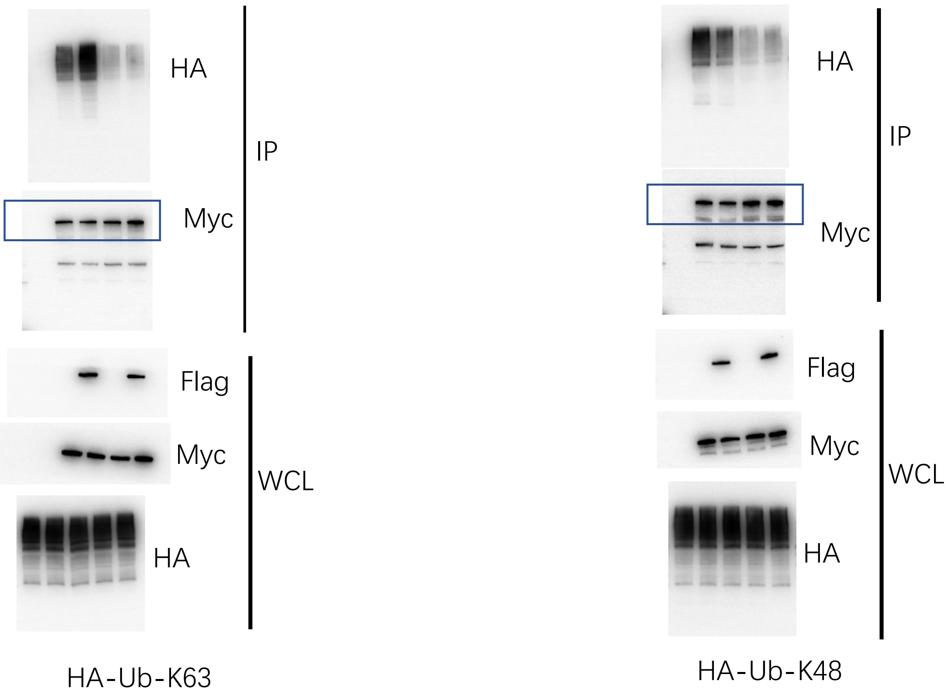

Fig 7G

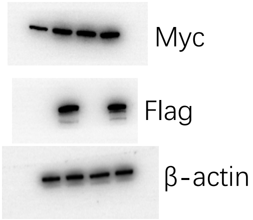

Supplementary Fig 4:

Supplementary Fig 4O:

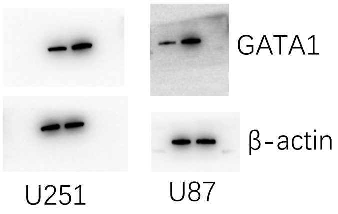

Supplementary Fig 4P:

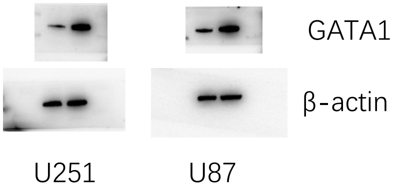

Supplementary Fig 4Q:

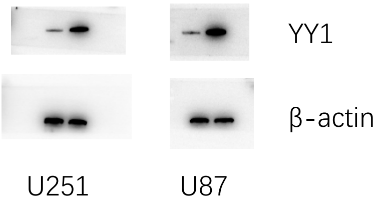

Supplementary Fig 4T:

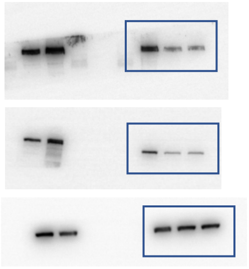

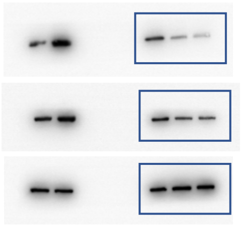

Supplementary Fig 4W:

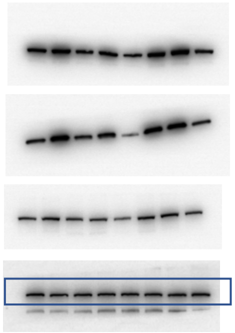

Supplementary Figure 7

Supplementary Figure 7A

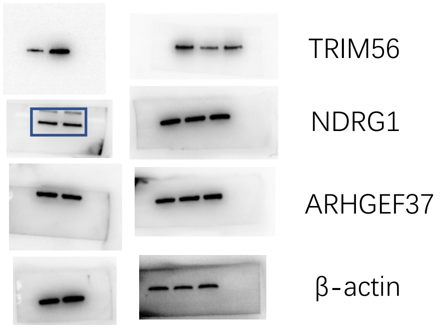

Supplementary Figure 7B

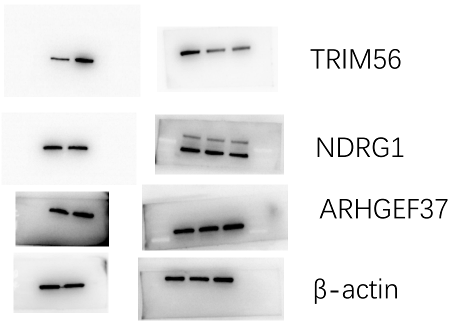

Supplementary Figure 8

Supplementary Figure 8B

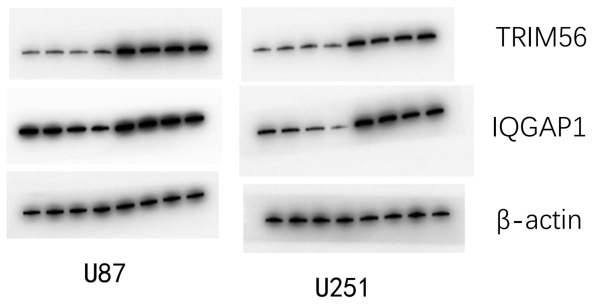

Supplementary Figure 9

Supplementary Figure 9A

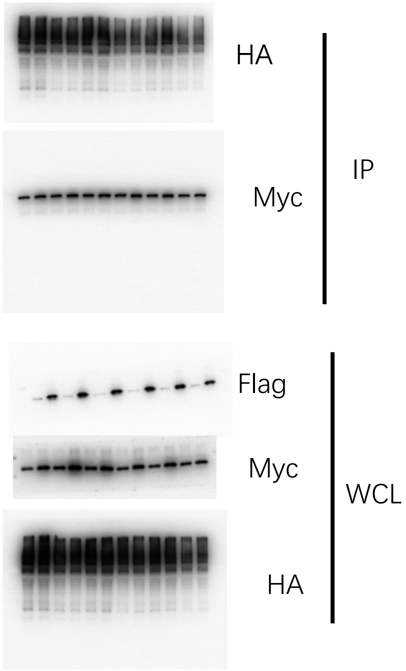

Supplementary Figure 9B

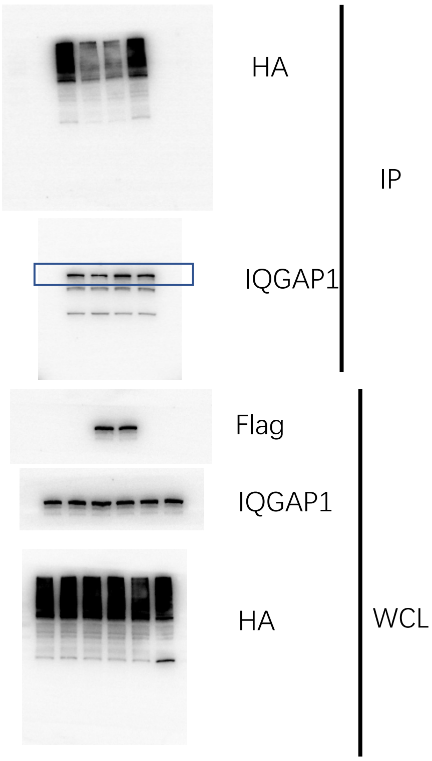

Supplementary Figure 9C

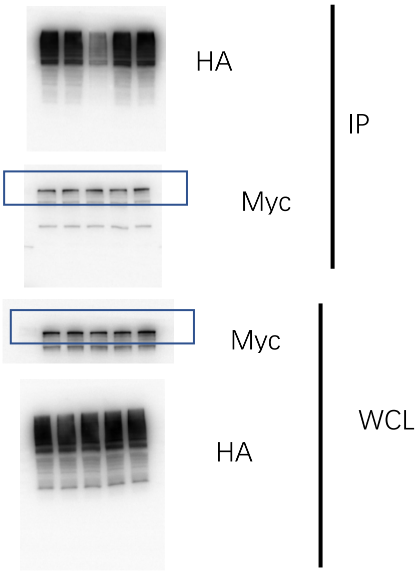

Supplement: Supplementary file 13 — Original western blots [file 41419_2023_5702_MOESM13_ESM.pdf]
